# MAPPING THE COMMONALITIES OF REHABILITATION: USING GUIDE-REHAB TO OPEN THE BLACK BOX OF PROGRAMMES ACROSS DIVERSE HEALTH CONDITIONS

**DOI:** 10.2340/jrm.v58.46211

**Published:** 2026-08-17

**Authors:** Stefano NEGRINI, Helena BURGER, Paolo CAPODAGLIO, Maria Gabriella CERAVOLO, Lisa HOOGENDAM, Esther JANSSEN, Greta JURENAITE, Calogero MALFITANO, Federico PENNESTRI, Ruud SELLES, Gianluca M. TARTAGLIA, Carlotte KIEKENS, the PREPARE Group

**Affiliations:** 1 Department of Biomedical, Surgical and Dental Sciences, University of Milan, Milan, Italy; 2 Laboratory of Evidence-Based Rehabilitation, IRCCS Galeazzi – Sant’Ambrogio Hospital, Milan, Italy; 3 University Rehabilitation Institute Republic of Slovenia, University of Ljubljana, Faculty of Medicine, Ljubljana, Slovenia; 4 IRCCS Istituto Auxologico Italiano, Milan, Italy; 5 Department of Experimental and Clinical Medicine, Marche Polytechnic University, Ancona, Italy; 6 Department of Rehabilitation Medicine, Erasmus MC, University Medical Center Rotterdam, the Netherlands; 7 Department of Plastic and Reconstructive Surgery, Erasmus MC, University Medical Center Rotterdam, the Netherlands; 8 IQ health, Radboud University Medical Center, Radboud Institute for Health Sciences, Nijmegen, the Netherlands; 9 School of Allied Health, HAN University of Applied Sciences, Nijmegen, the Netherlands; 10 Department of Orthopaedic Surgery, Viecuri Medisch Centrum, Venlo, the Netherlands; 11 ISICO (Italian Scientific Spine Institute), Milan, Italy; 12 Department of Biomedical Sciences for Health, University of Milan, Italy; 13 Azienda di Servizi alla Persona Istituti Milanesi Martinitt e Stelline e Pio Albergo Trivulzio, Milan, Italy; 14 Scientific Direction, IRCCS Galeazzi – Sant’Ambrogio Hospital, Milan, Italy; 15 Policlinico Hospital, Fondazione IRCCS Cà Granda Ospedale Maggiore Policlinico, Milan, Italy

**Keywords:** delivery of healthcare, International Classification of Functioning, Disability and Health, models, theoretical, patient care planning, qualitative research, rehabilitation

## Abstract

**Objective:**

Rehabilitation is a health strategy implemented with specific programmes across multiple health conditions. This study aimed to identify rehabilitation programmes’ commonalities among health conditions and partners, as required by the PREPARE (Personalised REhabilitation via Novel AI PAtient StRatification StratEgies) project.

**Design:**

Qualitative study.

**Subjects/Patients:**

Seven rehabilitation programmes in Europe covering diverse health conditions were chosen according to PREPARE research needs for their availability of large clinical databases.

**Methods:**

The Guideline for Interventions Description in Rehabilitation (GUIDE-Rehab) was used to describe targets, components, and ingredients of rehabilitation programmes. These elements were mapped to the International Classification of Functioning, Disability, and Health (ICF) domains.

**Results:**

The programmes’ interventions covered all ICF domains collectively, but not individually. Body functions and structures were the most frequent domains, whereas participation and contextual factors were underrepresented. Similar components (e.g., exercise, orthoses) were applied to different objectives depending on the underlying intervention theory. Pharmacological and surgical elements were also integral parts of rehabilitation programmes.

**Conclusion:**

We found differences and incomplete coverage of ICF domains in highly specialized programmes, incorporating organ-based and functioning-based approaches. Future studies should check (*i*) the generalizability and (*ii*) if results are justified by differences among health conditions or due to incomplete rehabilitation programmes/description.

## LAY ABSTRACT

Rehabilitation helps people regain their daily functioning after illness, injury, or surgery. However, rehabilitation programmes are often described in vague ways, making it difficult for clinicians to compare them and to know exactly how to use them. In this study, we applied a new tool (GUIDE-Rehab) to clearly describe and compare seven different rehabilitation programmes across Europe. We found that, while these programmes cover many areas, they often focus more on physical issues (such as pain and strength) and less on patients’ daily lives and social activities. We also observed that the same treatments, such as exercises, are used in different ways depending on the programmes’ goals. This work suggests that using a consistent, structured approach to describe rehabilitation can help clinicians choose and adapt the best treatments for their patients. In the future, digital tools may make this process even faster and easier to use in daily care.

Rehabilitation is a health strategy needed at all levels of care (primary, secondary, and tertiary) across the continuum of care and the lifespan (1). The World Health Organization (WHO) defines rehabilitation as “a set of interventions designed to optimise functioning and reduce disability in individuals with health conditions in interaction with their environment” (2). Cochrane Rehabilitation developed the following definition of rehabilitation for research purposes: “In a health care context”, rehabilitation is defined as a “multimodal, person-centred, collaborative process” (Intervention-general), including interventions targeting a person’s “capacity (by addressing body structures, functions, and activities/participation) and/or contextual factors related to performance” (Intervention-specific) with the goal of “optimising” the “functioning” (Outcome) of “persons with health conditions currently experiencing disability or likely to experience disability, or persons with disability” (Population) (3). These definitions clearly state the nature of rehabilitation as being cross-sectional to all health conditions. Nevertheless, the common characteristics of rehabilitation across multiple health conditions have seldom been evaluated.

The European-funded project “Personalized rehabilitation via novel AI patient stratification strategies” (PREPARE) (4) aims to advance rehabilitation care by using machine learning to analyse large-scale datasets, ultimately creating validated, data-driven prediction and stratification tools that help clinicians and patients select optimal therapy strategies. To achieve its objectives, PREPARE recruited 7 clinical rehabilitation groups from across Europe through an open call, each contributing a large dataset from a distinct health condition and sharing an interest in developing prediction models.

To address PREPARE’s aims, it was essential not simply to document the obvious differences among rehabilitation programmes targeting distinct health conditions, but to examine whether common structural principles underlie how individual rehabilitation plans are designed across diverse conditions. Although specific interventions vary according to patients’ different rehabilitation needs, it is less clear whether the clinical reasoning, organizational logic, and therapeutic principles guiding those prescriptions are consistent across programmes. Investigating these deeper commonalities and differences, beyond condition-specific treatment content, was therefore a key driver of this research. Nevertheless, identifying these commonalities and differences is not easy without an appropriate reporting tool. The reporting of rehabilitation interventions is a methodological issue (5). In clinical trials and observational studies, the quality of descriptions of interventions for rehabilitation is often poor, thus limiting their implementability, replicability, and the ability to build new studies (6). A general lack of transparency around the essential ingredients, methods of delivery, and hypothesized mechanisms of the effect of rehabilitation interventions in both experimental (7, 8) and clinical contexts (9) has cast a shadow over the quality of rehabilitation research. Recently, the “Guideline for Interventions Description in Rehabilitation” (GUIDE-Rehab) was developed (10) to support the reporting of complex rehabilitation interventions. PREPARE decided to adopt this reporting tool to open the black box of rehabilitation programmes. This study, nested within the PREPARE project, follows another that investigated whether rehabilitation variables across 7 large PREPARE partner databases share common characteristics (11).

The current paper examines the commonalities and differences among the 7 PREPARE rehabilitation programmes, which (*i*) address different health conditions, (*ii*) have been developed independently of one another, and (*iii*) span diverse countries. We compared the programmes using the GUIDE-Rehab and classified the listed targets, components, and ingredients according to the International Classification of Functioning, Disability and Health (ICF) domains. The final aims include the following research questions:

1. Are there similar characteristics of rehabilitation programmes proposed across diverse health conditions?
2. What can we learn from the implementation of GUIDE-Rehab in real-life clinical rehabilitation?

## METHODS

### Design

A qualitative study was conducted within the PREPARE project using an adapted graphical version of the GUIDE-Rehab tool to describe and compare interventions within rehabilitation programmes, including intervention theory, targets, components, and active ingredients, across 7 pilot cases. Each rehabilitation programme was described by the respective clinical partner using the GUIDE-Rehab tool, based on their clinical protocols and available data.

### Participants

The 7 rehabilitation programmes address heterogeneous health conditions requiring rehabilitation; they have been developed in different European countries, across multiple settings and for diverse age groups. Table I summarizes the clinical partners involved, and their programmes’ focus on a particular health condition, population, and setting. Further details are provided in Appendix S1 and in the paper on the first nested study (11).

**Table I:** List of clinical partners of the PREPARE project and the main characteristics of their rehabilitation programmes

N	Partner	Acronym	Health condition	Population age	Setting
1	Erasmus MC, Universitair Medisch Centrum Rotterdam, The Netherlands	EMC	People with hand and wrist disorders	Adults	Outpatient
2	ISICO (Istituto Scientifico Italiano Colonna Vertebrale), Milan, Italy	ISICO	People with idiopathic scoliosis	Children	Outpatient
3	Radboud Universitair Medisch Centrum, Nijmegen, The Netherlands	RAD	People with intermittent claudication	Adults	Outpatient
4	Univerzitetni Rehabilitacijski Inštitut Republike Slovenije Soča, Liubljana, Slovenia	URI	People with lower limb loss	Adults	Inpatient
5	Università Politecnica delle Marche, Ancona, Italy	UNIVPM	People with Parkinson’s disease or Parkinsonism	Adults (mostly older adults)	Outpatient
6	IRCCS Ospedale Galeazzi Sant’Ambrogio, Milan, Italy	OGSA	People who underwent hip and knee arthroplasty for osteoarthritis	Adults (mostly older adults)	Inpatient
7	Università degli Studi di Milano, Milan, Italy	UMIL	People with temporomandibular disorders	Adults	Outpatient

### Evaluation

**Fig. 1:**
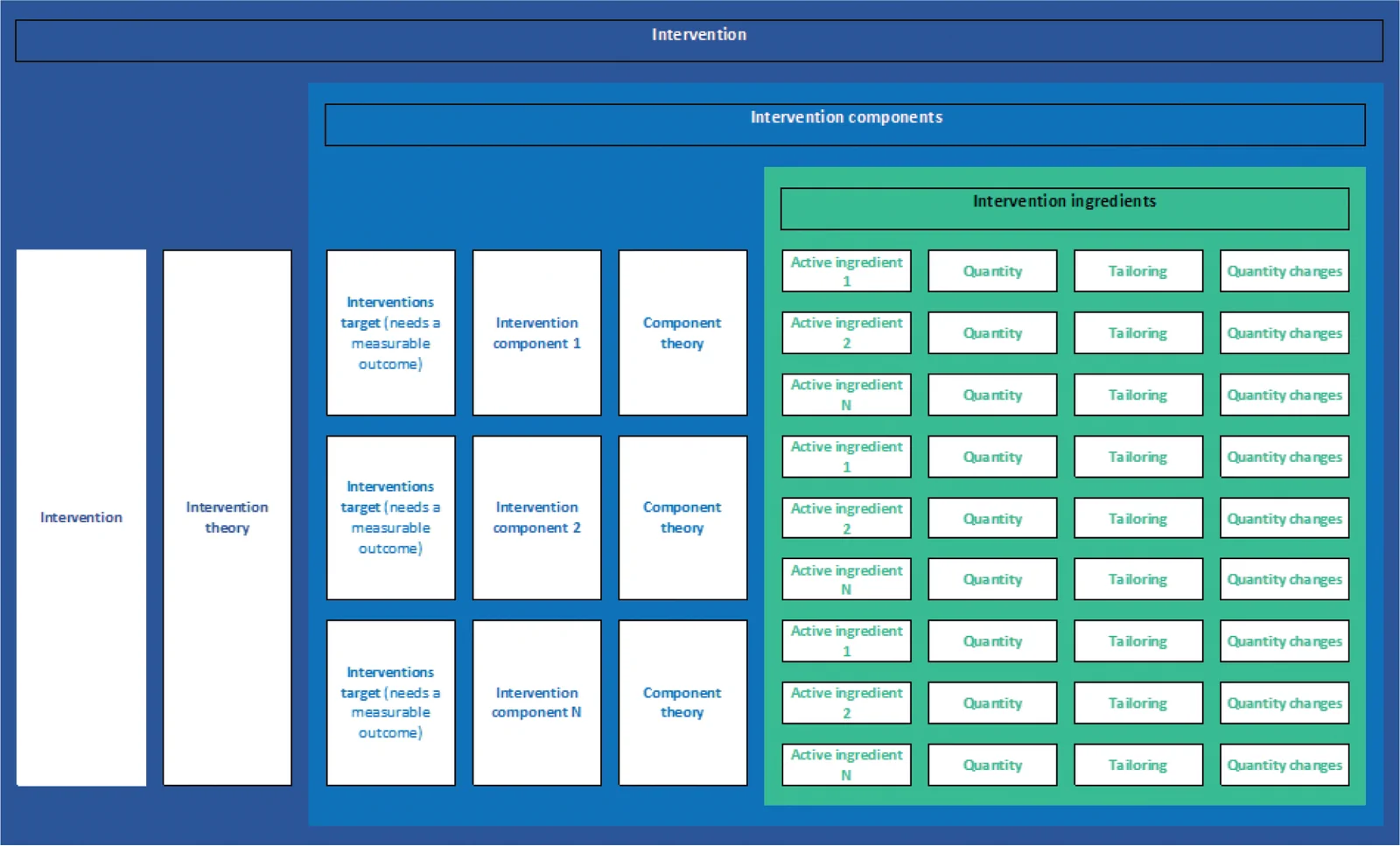
Graphic version of GUIDE-Rehab adapted for the PREPARE project.

The interventions within the 7 rehabilitation programmes were analysed and compared using an adapted graphical version of GUIDE-Rehab. Developed by Cochrane Rehabilitation, GUIDE-Rehab is a standardized reporting guideline designed for all rehabilitation study designs (10) and is registered on the Equator Network Library (12). While the full guideline includes 4 versions – ranging from the 16-item Complete version to the 9-item Graphical version – this study utilized a specific subset of items tailored to the PREPARE project. Specifically, we adopted items from the uncontrolled studies version that pertain exclusively to the articles’ methods section, omitting items related to the abstract, background, or results to focus strictly on intervention description. The specific items applied in this study are detailed in Table II, and the adapted graphic version is shown in Fig. 1.

**Table II:** Glossary of terms used in the GUIDE-Rehab reporting guideline. Only those used in the PREPARE project were inserted

Term	Definition
Intervention theory	The biological, psychological, and/or social reasons why the intervention should work in a specific situation
Intervention component	The set of active ingredients that accounts for change in a single intervention target
Component theory	The biological, psychological, and/or social reasons why the intervention should work in a specific situation
Intervention target	A measurable aspect of the functioning or personal factors hypothesized to be directly changed by the active ingredients of the intervention component
Active ingredient	A single constituent of the intervention responsible, alone or with other ingredients, for the therapeutic action

### Analysis

To compare the information collected in the GUIDE-Rehab graphs, we used the ICF categories (13). This was implemented for all the intervention targets of each individual rehabilitation programme. The targets were further aligned within each broader ICF category, and the treatment components followed the same categorization. The ingredients were then aligned with the categories of their respective components. A single researcher (SN) performed the categorization and alignments at all stages, and a second author (CK) checked them. At each stage, results were sent to the researchers responsible for each rehabilitation programme for verification and approval. Any discordance was resolved through discussion. This allowed a comparison of targets, components, and ingredients.

## RESULTS

The rehabilitation interventions and their respective theories are reported in Table III.

**Table. III:** Interventions and their respective theories of the PREPARE rehabilitation programmes

Partner	EMC	ISICO		RAD	UNIVPM			URI			OGSA	UMIL	
		Bracinga	Exercises		High intensity rehabilitation	Functional neurosurgery	Telerehabilitation	Falls prev-ention	Safe fitting and walking endurance with lower limb prosthesis	Preventing development of wounds on the residual limb		Irreversible	Reversible
Intervention	Nonsurgical treatment (i.e., hand therapy + splint for thumb base osteoarthritis)	Bracing rehabilitation treatment (includes exercises, cognitive-behavioural and psychological approach, sport activities and adapted therapeutic environment)	Physiotherapic scoliosis-specific exercises rehabilitation treatment (including exercises, cognitive-behavioural approach and sport activities)	Supervised exercise therapy for intermittent claudication (IC)	High intensity multimodal training (HIT)	Functional neurosurgery for deep brain stimulation	Telerehabilitation (TR)	After lower limb loss (LLL), all patients are referred to our outpatient clinic and admitted for comprehensive rehabilitation. At admission, we take the patient’s history and perform a clinical examination, and all team members do extensive testing in the first three days. We discuss the results at our first team meeting and decide about further interventions. We follow all internationally published guidelines.			Inpatient rehabilitation after total hip or knee replacement for osteoarthritis	Irreversible therapy in temporomandibular disorder (TMD) management involves invasive or surgical interventions that result in permanent alterations to the temporomandibular joint (TMJ) or surrounding structures. These interventions are typically considered when conservative treatments fail to provide adequate relief, or when structural abnormalities or pathology require more aggressive management.	Reversible therapy in TMD management typically involves non-invasive or conservative treatments aimed at alleviating symptoms and addressing underlying factors contributing to TMD. These therapies focus on reversible interventions that do not permanently alter the structure or function of the TMJ or surrounding tissues.
Intervention theory	First carpometacarpal joint (CMC-1) osteoarthritis (OA) leads to synovitis, instability, and varying degrees of hyperextension of the first meta-carpophalangeal joint. The first part of this intervention is intended to reduce synovitis, and the second part is to improve the thumb’s active stability to prevent subluxation and further degeneration of the CMC-1 joint.	Braces immediately improve the pathological curve and change the asymmetrical compression of the vertebrae. Their use until bone maturity may allow vertebrae to undergo corrective remodelling. Maximal correction is achieved in the first months of treatment; the remaining treatment focuses on maintaining the correction. Exercises and gradual brace weaning (to gradually adapt the trunk and spine to doffing without losing correction) play a major role in maintaining the results. Gradual brace weaning also provides a rewarding system (brace reduction at each consultation) to keep compliance.	Physiotherapic scoliosis-specific exercises teach active self-correction and challenge it through different movements and stabilisation exercises that aim to maintain the self-correction over time. The goal is to develop an automatic counter-scoliosis postural support and movement, enhancing neuromotor capacity and strength-ening spinal stability in self-correction. A cognitive-behavioural approach supports psychological well-being and promotes compliance. Sports activities reinforce this effect and offer psychological benefits. Motivational tools are available on a website and other resources. Regular or on-call emails, calls, video consults, and meetings are used as needed.	Evidence shows Treadmill training is effective for increasing walking distance and functional ability in people IC. For other treatment inter-ventions there is less evidence available. However, through functional training, lifestyle interventions and improvement of self-management patients are expected to be able to better independ-ently manage their health and improve their quality of life. In general conservative care is more cost-effective than surgical treatment.	High-intensity multimodal training aims to increase cardiorespiratory fitness, overcome psychological restraints preventing people from engaging in regular physical activity, promote independence in activity of daily living by targeting different functions which may be impaired in people with Parkinson’s disease (PD) or Parkinsonism (namely, speech, hand dexterity, balance)	Deep brain stimulation of subthalamic nuclei is an established effective approach to the symptomatic treatment of cardinal motor features in people with PD. Specifically, the activation of electromagnetic fields within the subthalamus produces a modulation of the cortical-thalamic-striatal pathway, able to achieve the same improvement in the cardinal motor features (i.e. rest tremor, rigidity, bradykinesia) as the optimal levoDopa dose.	Telerehabilitation (TR) enables training for longer periods in real-world scenarios. TR can be highly motivating and challenging, increasing adherence to treatment. Access to specialised care can be difficult, and TR, together with telemonitoring, is a viable option for patients who require an ongoing, long-term approach. The economic and human burden of PD care is high, and TR is proposed as a tool to ensure personalised, equally distributed, continuous training.	Within the multimodal training it is expected that educationin of proper execution of ADL and removal of barriers in home environment, balance training and use of appropriate wallking aids significantly reduce number of falls			Pharmacological pain treatment is prescribed to help patients recovery after surgery. Deep Venous Thrombosis prevention is prescribed by means of pharmacological and mechanical means to prevent cardiovascular complications after surgery. The remaining exercises (joint and muscle strength recovery, postural steps and transfers trainings, climbing stairs, avoiding risky postures and movements, ADL) are prescribed to recover the maximum extent of autonomy before discharge and achieve global and segmental functional recovery.	Irreversible therapy targets structural changes in the TMJ for long-lasting TMD relief. These methods carry higher risks and longer recovery than reversible treatments, requiring careful, personalized planning. Examples include: TMD Arthrocentesis, a minimally invasive irrigation and aspiration to reduce inflammation; arthroscopy or open-joint surgery, which allow visualization and repair of issues like adhesions, disc displacement, or synovial problems; and joint replacement with prosthetics in severe TMJ degeneration or dysfunction.	Reversible therapay alleviates symptoms and functional limitations without invasive procedures or permanent TMJ alterations. It includes: self-management techniques like exercises, stretches, and manual therapies to improve jaw mobility, reduce muscle tension, and enhance TMJ function; occlusal splints that distribute forces, lessen clenching or grinding, and provide temporary pain relief; pharma-cotherapy options like NSAIDs, muscle relaxants, or tricyclic anti-depressants for pain, inflammation, or issues like bruxism or anxiety; and behavioral therapies such as stress management and relaxation techniques.

EMC: Erasmus MC, Universitair Medisch Centrum, Rotterdam, The Netherlands; ISICO; Istituto Scientifico Italiano Colonna Vertebrale, Milan, Italy; RAD: Radboud Universitair Medisch Centrum, Nijmegen, The Netherlands; UNIVPM: Università Politecnica delle Marche, Ancona, Italy; URI: Univerzitetni Rehabilitacijski Institut Republike Slovenije-Soca, Ljubljana, Slovenia; OGSA: IRCCS Ospedale Galeazzi Sant’Ambrogio, Milan, Italy; UMIL: Università degli Studi di Milano, Milan, Italy.

**Fig. 2:**
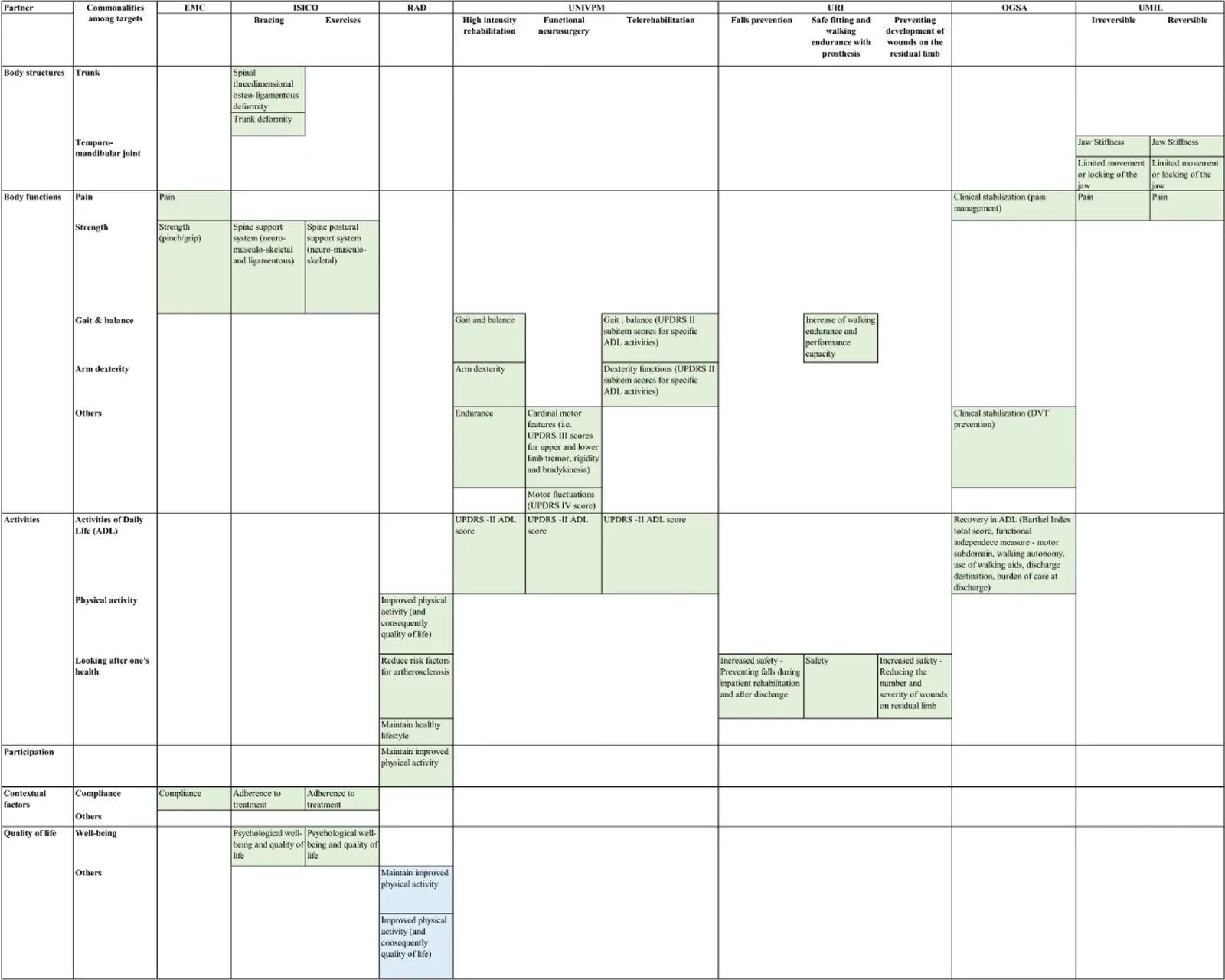
Intervention targets indicated by the PREPARE clinical partners of interventions according to the ICF categories. Some targets that spanned more than 1 category are highlighted in light blue. EMC: Erasmus MC, Universitair Medisch Centrum, Rotterdam, The Netherlands; ISICO; Istituto Scientifico Italiano Colonna Vertebrale, Milan, Italy; RAD: Radboud Universitair Medisch Centrum, Nijmegen, The Netherlands; UNIVPM: Università Politecnica delle Marche, Ancona, Italy; URI: Univerzitetni Rehabilitacijski Institut Republike Slovenije-Soca, Ljubljana, Slovenia; OGSA: IRCCS Ospedale Galeazzi Sant’Ambrogio, Milan, Italy; UMIL: Università degli Studi di Milano, Milan, Italy.

The targets of the interventions considered by the PREPARE clinical partners span the full range of ICF categories; however, none of the partners addresses all categories simultaneously (Fig. 2). Body Structures are most often addressed in programmes focusing on specific areas of the body (e.g., the spine and the temporomandibular joint). Six out of seven programmes included targets related to Body Functions. Across these programmes, pain was targeted in three programmes, strength-related impairments in two, gait and balance in two, arm dexterity in one, and other body functions in one. Several programmes addressed more than one Body Functions target. Three programmes target Activities: 2 target ADL and self-management, and 1 physical activity. Participation is explicitly considered by 1 partner, and indirectly in terms of “maintenance of improved physical activity (and consequently participation)”. Two partners considered Contextual Factors. Additionally, quality of life cannot be categorized in the ICF but was addressed by interventions in 2 programmes targeting psychological well-being.

**Fig. 3:**
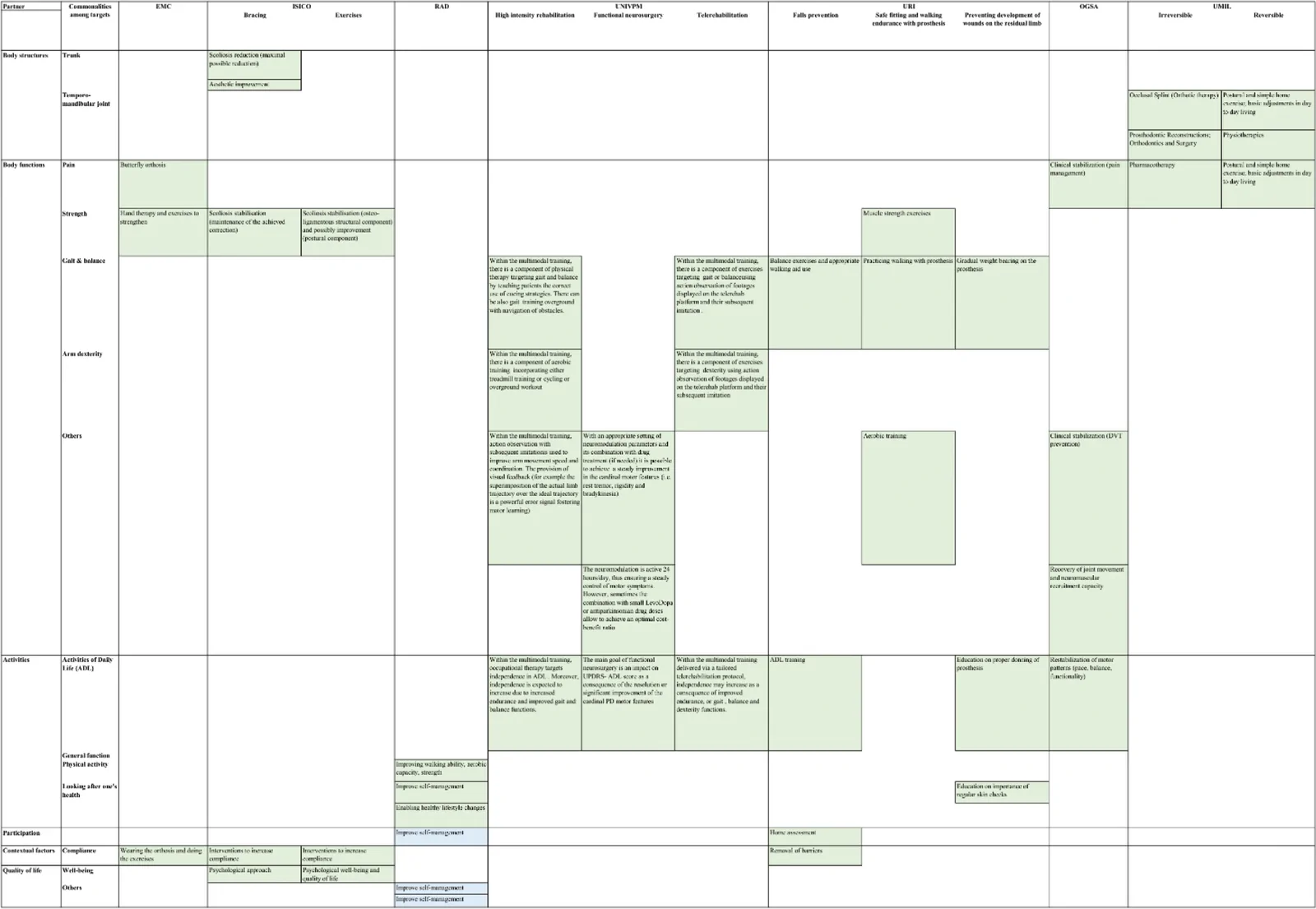
Intervention components categorized by target. Some targets that spanned more than 1 category are highlighted in light blue. EMC: Erasmus MC, Universitair Medisch Centrum, Rotterdam, The Netherlands; ISICO; Istituto Scientifico Italiano Colonna Vertebrale, Milan, Italy; RAD: Radboud Universitair Medisch Centrum, Nijmegen, The Netherlands; UNIVPM: Università Politecnica delle Marche, Ancona, Italy; URI: Univerzitetni Rehabilitacijski Institut Republike Slovenije-Soca, Ljubljana, Slovenia; OGSA: IRCCS Ospedale Galeazzi Sant’Ambrogio, Milan, Italy; UMIL: Università degli Studi di Milano, Milan, Italy.

The intervention components and ingredients, organized by target organization, are shown in Fig. 3.

Notably, the same treatments are implemented to achieve different targets depending on the context. Orthoses are used for pain and body structures, while exercises address body structures, pain, functions, and activities. Other interventions target broader domains: self-management addresses participation, while specific interventions focus on quality of life, psychological well-being, and compliance. Additionally, some interventions that would typically not be classified as rehabilitation – such as pharmacotherapy for pain and surgical procedures targeting body structures or functions – are reported as an integral part of the rehabilitation process.

## DISCUSSION

This study, conducted within the PREPARE project, applied an adapted graphical version of GUIDE-Rehab to describe and compare 7 heterogeneous rehabilitation programmes in Europe. The findings show that, while intervention targets collectively span the full range of ICF domains, no single programme covers all ICF categories.

These results reflect current clinical practice, where rehabilitation programmes often prioritize impairment-based targets despite the broader biopsychosocial framework of the ICF (1, 3). For instance, GUIDE-Rehab reveals that while “Participation” (e.g., social life) is the ultimate goal of the ICF philosophy (14), it was the least-represented target. This limited representation together with that of contextual factors may indicate challenges in operationalizing these domains in routine care. At the same time, similar intervention components (e.g., exercise, orthoses, prostheses, self- management) were applied to achieve different therapeutic targets, highlighting the flexibility of rehabilitation strategies in line with their underlying rationale. For instance, “exercise” serves as a wildcard intervention depending on the logic described in GUIDE-Rehab: it may target Body Structures (e.g., scoliosis correction) or Activities (e.g., walking distance in claudication). Moreover, in the rehabilitation process, we identified not only classical interventions (e.g., therapeutic exercises, orthoses, physical modalities, and other interventions) and relevant variables (e.g., treatment timing and medical history), but also treatments such as drugs, surgery, or prevention. Those are not typically considered rehabilitation when classified as a “non-pharmacological, non-surgical” approach. They also fail to meet the definitions of “rehabilitation is what rehabilitation professionals do” (15) or the WHO definition (2), because some of these interventions are not practised within rehabilitation services. Nevertheless, they can be part of the rehabilitation process, such as pharmacotherapy for pain and surgical procedures for hand and wrist function, in line with the Cochrane Rehabilitation definition of rehabilitation (3).

The answer to the research question on similarity of rehabilitation programmes proposed across diverse health conditions is somehow dubious. While, on the one hand, it could be said that there are no clear-cut common pathways for implementing rehabilitation as a common strategy, the results of this study also support the characterization of rehabilitation as a cross-sectional strategy across diverse health conditions. The 7 studied programmes collectively cover the full range of ICF domains. Although no single programme addressed all ICF categories, the combined scope of these programmes demonstrates that rehabilitation interventions can be unified under a single, comparable structure regardless of the specific pathology. This cross-sectional nature is further highlighted by the versatile application of similar intervention components for different therapeutic targets based on the specific needs of the patient population, illustrating how shared methodologies are applied across heterogeneous clinical cases. Ultimately, while specialized programmes prioritize different impairments, they utilize common components and ingredients that span the entire biopsychosocial framework of health.

Our study highlights a functional linkage between GUIDE-Rehab and ICF in clinical practice. While the ICF provides a framework, GUIDE-Rehab operationalizes it for intervention planning by mapping intervention targets to specific ICF components (Body Functions, Body Structures, Activities, Participation, Contextual Factors). It provides granular descriptions of the therapeutic intents that standard ICF codes often fail to capture. By requiring an explicit “Intervention Theory” (e.g., how bracing mechanically alters vertebral growth in scoliosis), GUIDE-Rehab unveils the causal link between the intervention ingredient (the orthosis) and the ICF outcome (structural stability). Moreover, cultural and language considerations may impact the consistent and accurate understanding of, for example, the intuitive descriptions of the ICF categories developed for the ICF Generic-30 Set (16, 17). The implementation of GUIDE-Rehab might mitigate this cultural inconsistency in ICF coding by requiring researchers to be explicit regarding the ingredients, thereby reducing ambiguity. Forcing a granular definition of ingredients and theories, GUIDE-Rehab could provide the missing link between clinical activities and the ICF. Using GUIDE-Rehab could also help verify whether clinicians actually deliver interventions that align with their stated ICF targets and result in changes in ICF participation codes or revert to impairment-based care.

Beyond the conceptual utility of GUIDE-Rehab, significant gaps remain in its practical implementation. Our study focuses on the output of the reporting tool but glosses over the effort required for input. Pilot feedback indicated that using GUIDE-Rehab was “difficult and time-consuming”. Future research must assess the implementation burden in routine clinical settings, including whether GUIDE-Rehab can be integrated into Electronic Health Records via Natural Language Processing (NLP) to automate ingredient extraction and mapping to ICF codes, in line with emerging trends in digital health.

Practical implications of our study are threefold. First, routine use of GUIDE-Rehab would increase transparency and replicability of rehabilitation interventions, facilitating evidence synthesis and implementation. Second, the combined GUIDE-Rehab/ICF approach provides an operational bridge between granular intervention descriptions and standardized functional coding, which is valuable for designing interoperable databases and for informing machine-learning models for personalised rehabilitation. Third, implementation challenges – chiefly the time and expertise required to complete GUIDE-Rehab – must be addressed through user training, streamlined workflows, and technological supports such as integration into electronic health records and automated extraction via natural language processing. Ultimately, adopting standardized reporting is a prerequisite for elevating rehabilitation to a high-quality, evidence-based pillar of universal health coverage.

### Limitations

Limitations of this work include its pilot nature, the small and heterogeneous sample of rehabilitation programmes from 7 clinical partners within the PREPARE project, the use of a restricted subset of GUIDE-Rehab items (excluding quantities and tailoring), and the lack of direct linkage to patient outcomes or to inter-rater reliability testing. Furthermore, this work was performed also to pilot the new GUIDE-Rehab instrument in everyday clinical life. Imprecisions in answering the requests or incomplete understanding of the guidelines could also explain part of the results. Moreover, there could be difficulties in collecting some data in specific settings or due to the lack of validated outcome measures. Future research should (*i*) evaluate GUIDE-Rehab’s feasibility and burden in routine clinical practice, (*ii*) expand its application across larger and more diverse datasets, (*iii*) incorporate quantities, tailoring, and fidelity assessments, and (*iv*) connect detailed intervention descriptions to outcomes to enable robust development and validation of AI-driven prediction and stratification tools. Addressing these steps will help translate detailed intervention descriptions into better personalized rehabilitation care and stronger evidence for policy and practice. From a content perspective, future studies should check (*i*) the generalizability of these results and (*ii*) if the incomplete coverage of ICF domains is justified by differences among health conditions or is due to an incomplete rehabilitation description/approach of the programmes.

### Conclusions

This study demonstrates that the GUIDE-Rehab reporting guideline provides a robust and granular framework for describing complex rehabilitation interventions. By applying this tool across the 7 diverse rehabilitation programmes included in the PREPARE project, we successfully mapped heterogeneous clinical practices – ranging from surgical recovery to chronic disease management – into a unified, comparable structure.

Our findings highlight a critical synergy between GUIDE-Rehab and the ICF framework. While the ICF offers a standardized language for health outcomes, GUIDE-Rehab provides the necessary “missing link” by making intervention theories and active ingredients explicit. This transparency is essential for the development of the AI-driven stratification and prediction models that the PREPARE project aims to provide. However, while GUIDE-Rehab improves scientific rigour and replicability, its manual application is time-consuming. To scale this level of reporting across global health systems, future research should focus on automating the extraction of these clinical “ingredients” using Natural Language Processing (NLP) and integrating them with electronic health records.

## Supplementary Material


