# Supplementary material for: MAPPING THE COMMONALITIES OF REHABILITATION: USING GUIDE-REHAB TO OPEN THE BLACK BOX OF PROGRAMMES ACROSS DIVERSE HEALTH CONDITIONS
Source: J Rehabil Med. 2026 Aug 17;58:46211. doi: 10.2340/jrm.v58.46211 (PMC13488149; doi:10.2340/jrm.v58.46211)
Supplement: Supplementary file 1 [file JRM-58-46211-s1.pdf]

Appendix S1

Descriptions and intervention theories

Programme 1: rehabilitation for people with hand and wrist disorders (Partner EMC)

Overall description: The intervention targets nonsurgical management of thumb base osteoarthritis through splinting and exercises. Following interdisciplinary discussion (hand surgeon, hand and occupational therapists), patients receive a custom-made thermoplastic or neoprene orthosis. In the initial phase (weeks 0–6), the orthosis is worn continuously, except during exercises, which focus on coordinating specific thumb muscles. In the second phase (weeks 6–12), orthosis use is gradually reduced, and strengthening exercises are introduced to improve CMC-1 stability and reduce synovitis. The orthosis positions the thumb in CMC-1 extension and abduction, and MCP-1 flexion. The exercise program, delivered both in-clinic and at home, focuses on enhancing active stability of the CMC-1 during pinch in extension and abduction. The number of exercises can vary based on the therapist and the patient's considerations, such as therapy costs or travel distance (16).
Intervention theory: CMC-1 OA leads to synovitis of the CMC-1 joint, instability of the CMC-1 joint, and MCP-1 hyperextension in varying degrees. The first part of this intervention is meant to reduce synovitis. The second part is to improve the active stability of the thumb to prevent subluxation and further degeneration of the CMC-1 joint.

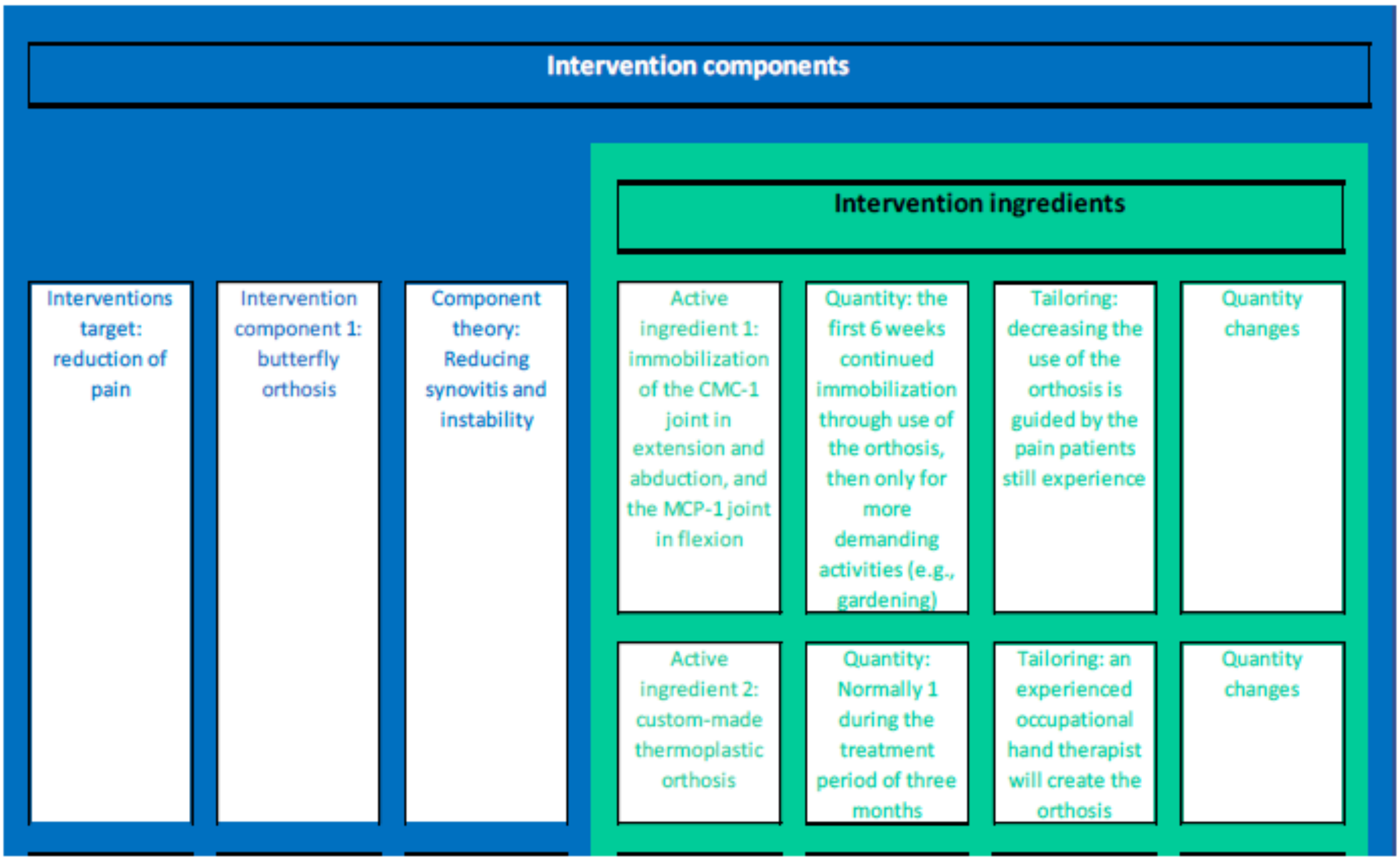

Figure 1 GUIDE-Rehab Programme 1 component 1

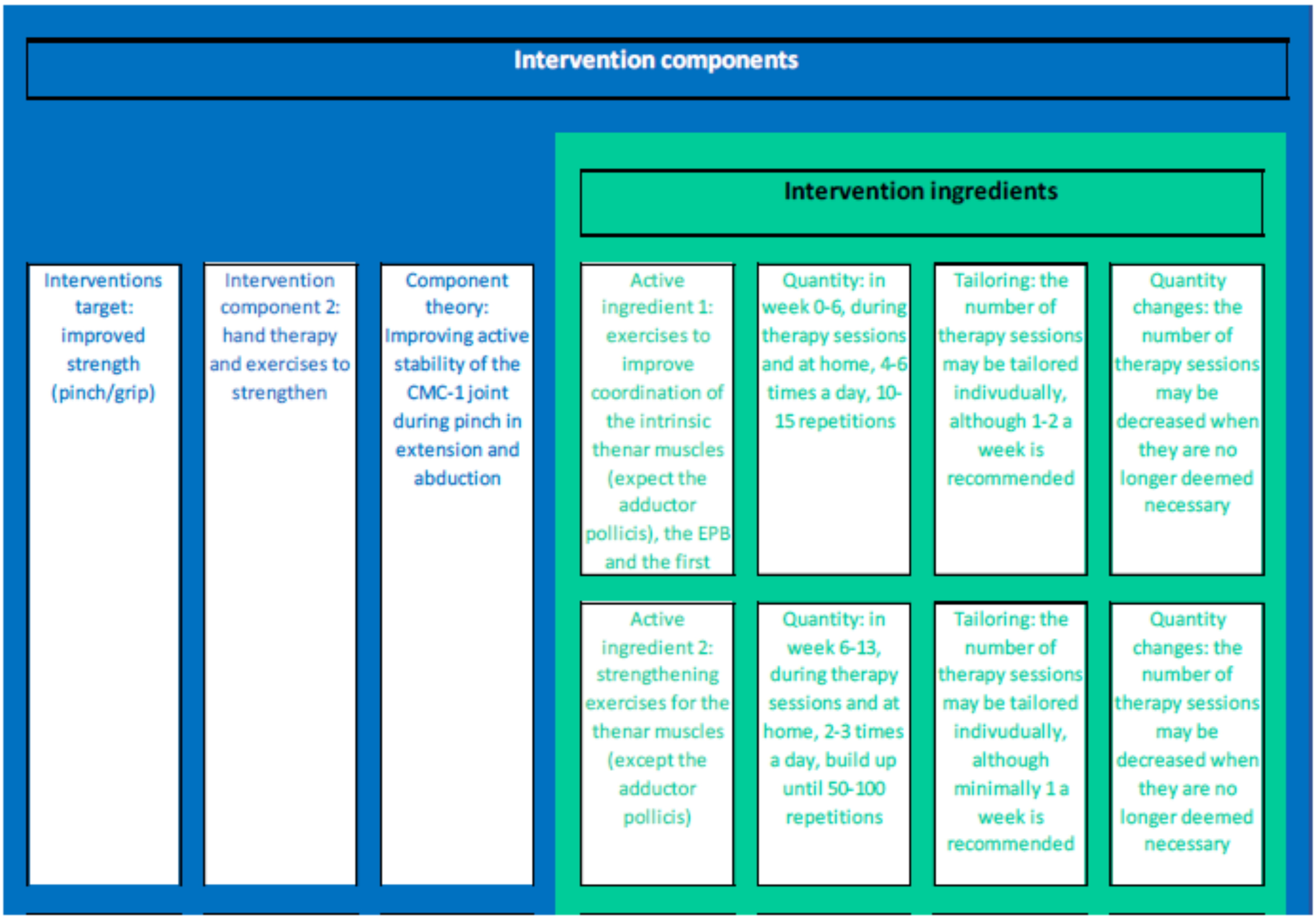

Figure 2 GUIDE-Rehab Programme 1 component 2

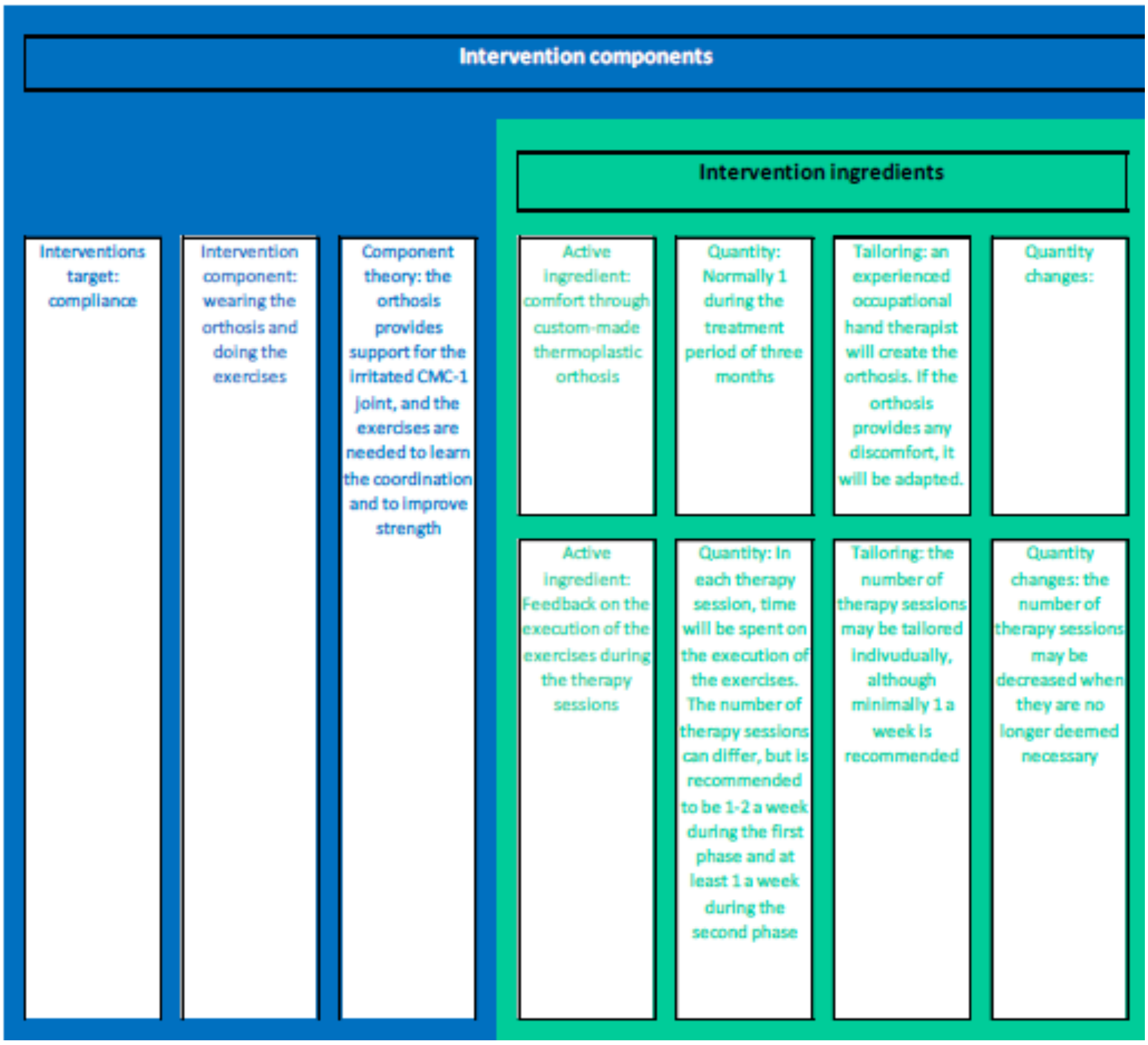

Figure 3 GUIDE-Rehab Programme 1 component 3

Programme 2: rehabilitation for children with idiopathic scoliosis (Partner ISICO)

The considered clinical scenarios include: 1) brace treatment (including physiotherapeutic scoliosis-specific exercises, PSSE), or 2) PSSE alone.

1) Overall description (braces): Braces are proposed with PSSE, cognitive behavioural approach, exercises, psychological approach, sports activities and an adapted therapeutic environment. It is intended to achieve radiographic results that are compatible with the spine's good functioning. The goal of brace treatment varies according to the degree of curvature considered, and forces (in terms of strength of brace and hours of usage) are consequently administered. The following extreme cases need to be considered: 1) In mild progressive adolescent scoliosis (up to 30° Cobb angle) that cannot be controlled through PSSE, the first aim is to avoid progression while allowing the maximum possible freedom in activities of daily life and reducing the discomfort caused by the brace; 2) In severe adolescent scoliosis (up to 45°-50° Cobb angle, and more if the patient doesn't want surgery or if surgery is not possible), the aim is at least to avoid progression (and surgery) and possibly also to reduce the amount of curvature, which does not guarantee stability in adulthood. In these cases, a brace is worn all day long for at least one year; 3) In juvenile and infantile scoliosis over 25°-30° Cobb angle, a brace is proposed for at least 18 hours/day according to the degree of curvature. When a result is obtained, the brace is progressively weaned with the aim of taking it off, possibly before the pubertal growth spurt. In all other cases, treatment is tailored according to individual preferences, anthropometric characteristics and other risk factors such as rotation, hump, lumbar curve take-off, unbalance, etc. Treatment is carried out by wearing the brace full-time until the period of rapid growth is over, and we foresee not having to face other pushes of the pathology. This is usually achieved at Risser stage 3. The applied full-time concept is again tailored between 18 and 23 hours/day, where 18 is considered a reasonable compromise between efficacy (full-time wearing 21 to 23 hours per day) and

acceptability (e.g. school, without a brace) with the goal of obtaining compliance. In this period, it is possible to start a partial weaning (increasing the hours without the brace while allowing the patient to maintain the achieved correction). Braces place the spine in the best possible correction and cause it to grow in this position. Whenever the brace is taken off, correction is progressively loosened, and the daily number of hours without the brace determines the real result achieved in that stage of treatment. Each time the spine regresses from the maximum correction, the vertebrae are again pushed toward deformity. This explains why the maximum correction achieved through bracing is never maintained and why weaning the brace for a longer than usual period can cause pain. We reduce brace wearing by no more than two or three hours every six months.

Intervention theory (braces): Braces mechanically improve the pathological curve and change the asymmetrical compression of the vertebrae. Their use till bone maturity possibly allows vertebrae corrective remodelling. Maximal correction is achieved in the first months of treatment, the remaining treatment focuses on keeping the correction. Exercises and gradual brace weaning (to gradually adapt the trunk and spine to doffing without losing correction) play a major role in maintaining the results. Gradual brace weaning also provides a rewarding system (brace reduction at each consultation). Exercises also reduce the brace's side effects, including reduced anti-gravity neuromuscular trunk capacity and spine rigidity. They also increase the patient's counter-scoliosis neuromotor capacity (reduction of the postural collapse on the deformity). A cognitive-behavioural approach, motivational tools, an adapted therapeutic environment, and psychological support help maintain well-being and quality of life and allow patients to achieve the best adherence possible. Physical activities potentiate the effects of other treatments and have psychological value.

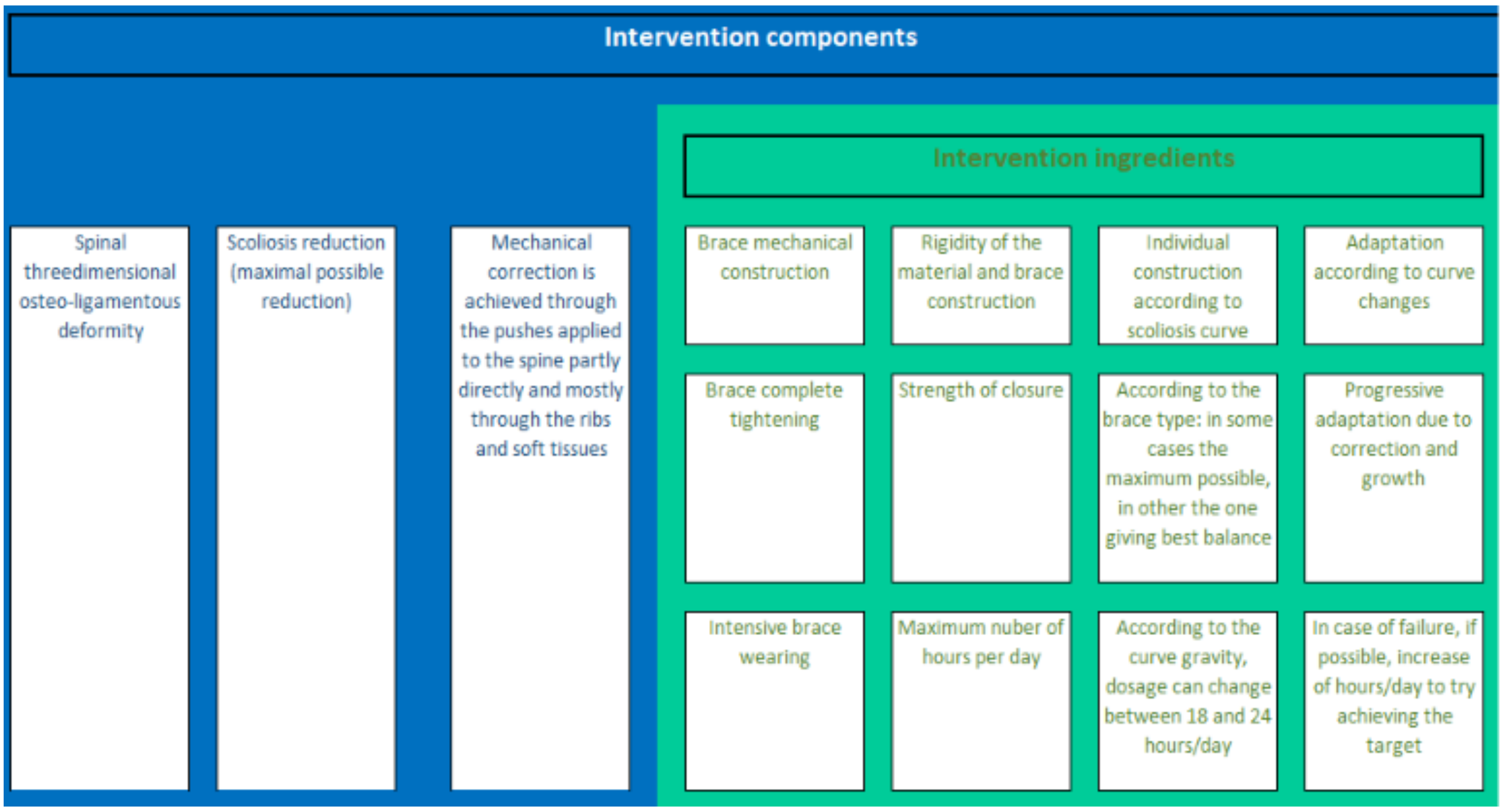

Figure 4 GUIDE-Rehab Programme 2 component 1 for bracing rehabilitation treatment

Intervention components

			**Intervention ingredients**			
Spine support system (neuro-musculo-skeletal and ligamentous)	Scoliosis stabilisation (maintenance of the achieved correction)	Maximum possible maintenance of the maximal correction is achieved with time and adequate length of treatment; mechanically, through progressive bone, discs and ligaments maturation and remodelling (structural correctable component of scoliosis); posturally through neuro-muscular anti-gravity and anti-scoliosis reprogramming (postural component of scoliosis). Sports activities provide increased neuro-motorial capacity to build-up with specific exercises	Prolonged brace wearing	Till the end of growth	According to scoliosis starting severity, achieved results , shared-decisions and adherence achieved and possible	Individually adapted according to scoliosis starting severity, achieved results, shared-decisions and adherence achieved and possible. At final weaning, if failure (progression >3° at x-ray after 48 hours without the brace) treatment
			Constant daily use of the brace	Doffing always of the same duration everyday	Some small adaptations are possible to facilitate compliance according to the curve gravity	During the summer, 1-2 hours more doffing for 2-4 weeks according to the results. Not done at the start of
			Gradual brace weaning	Additional doffing hours/day	Individually adapted according to results and compliance	2 hours/day additional doffing every 6 months (exception: from 24 and 23 hours/day, 1 hour/day doffing) keeping at least 18 hours/day till European Risser
			Active self-correction witohut external aids (including without the brace)	Introduced in all scoliosis specific exercises performed 20 min/day, 6 times/week	Individually adapted according to the neuromotor capacities	N/A
			Stability exercises without the brace	20 minutes/day; 6 days/week	Individually adapted according to capacity	Difficulty of exercises gradually increased with learning (training) and inmproved patients' ability
			Introduction of self-correction in everyday activities without the brace	As much as possible	Gradually introduced while learning and increasing neuromotor control	N/A
			Sports/Physical activity	At least 2 sessions per week added to the school	According to patients' preferences	According to patients' preferences

Figure 5 GUIDE-Rehab Programme 2 component 2 for bracing rehabilitation treatment

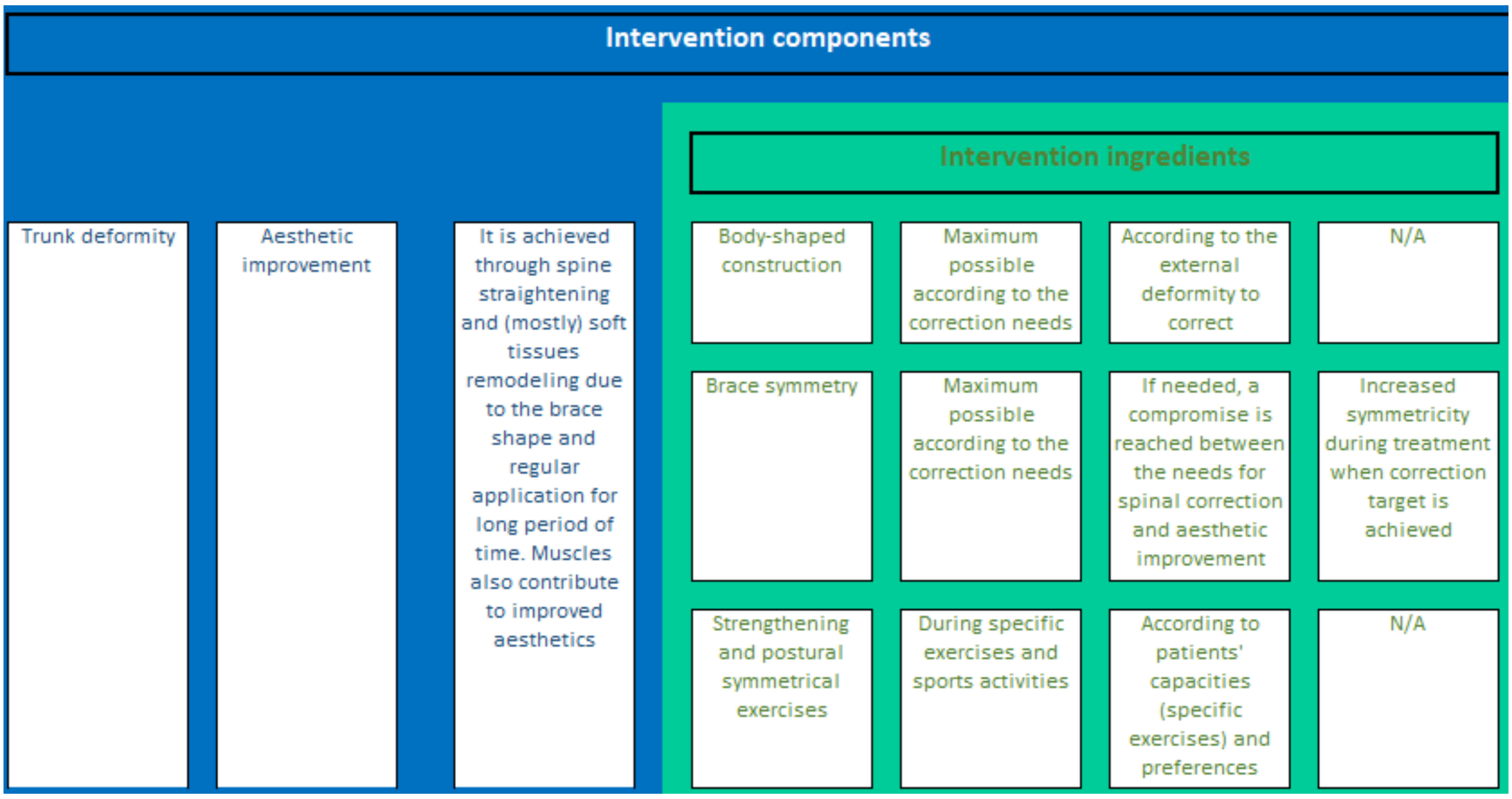

Figure 6 GUIDE-Rehab Programme 2 component 3 for bracing rehabilitation treatment

Intervention components						
			Intervention ingredients			
Adherence to treatment	Interventions to increase compliance	Adherence to treatment is based on the complete involvement of the patient and family. External support is provided at some key moments and on demand. A key role is also in how the brace is built and delivered. Multiple tools can help. All the instruments meant to keep well-being and quality of life also contribute to increase adherence, and vice-versa.	Shared decision making	As much as possible	Always	N/A
			Brace invisibility and comfort	As much as possible	The best possible compromise among correction need, invisibility and comfort is always searched with priorities set case-by-case	N/A
			Medical brace check for invisibility, comfort and correction	At each new brace delivery	Always	N/A
			Motivational talks at each encounter with professionals	Specific interventions at first prescription (15-20 minutes by physician; 30-45 minutes by physiotherapist), and at first brace delivery (10-15 minutes by physician). Always devoted some time at all other professional encounters	Always	According to the individual need to prevent or counteract adherence drop/loss
			Compliance tools: compliancemeter, graph comparing results with natural history, patient's trunk photographs and radiograph images pre/post treatment, movies and pieces of art from the "Concorsetto" (Brace Contest Award)	Individually used according to the need	Always	N/A

Figure 7 GUIDE-Rehab Programme 2 component 4 for bracing rehabilitation treatment

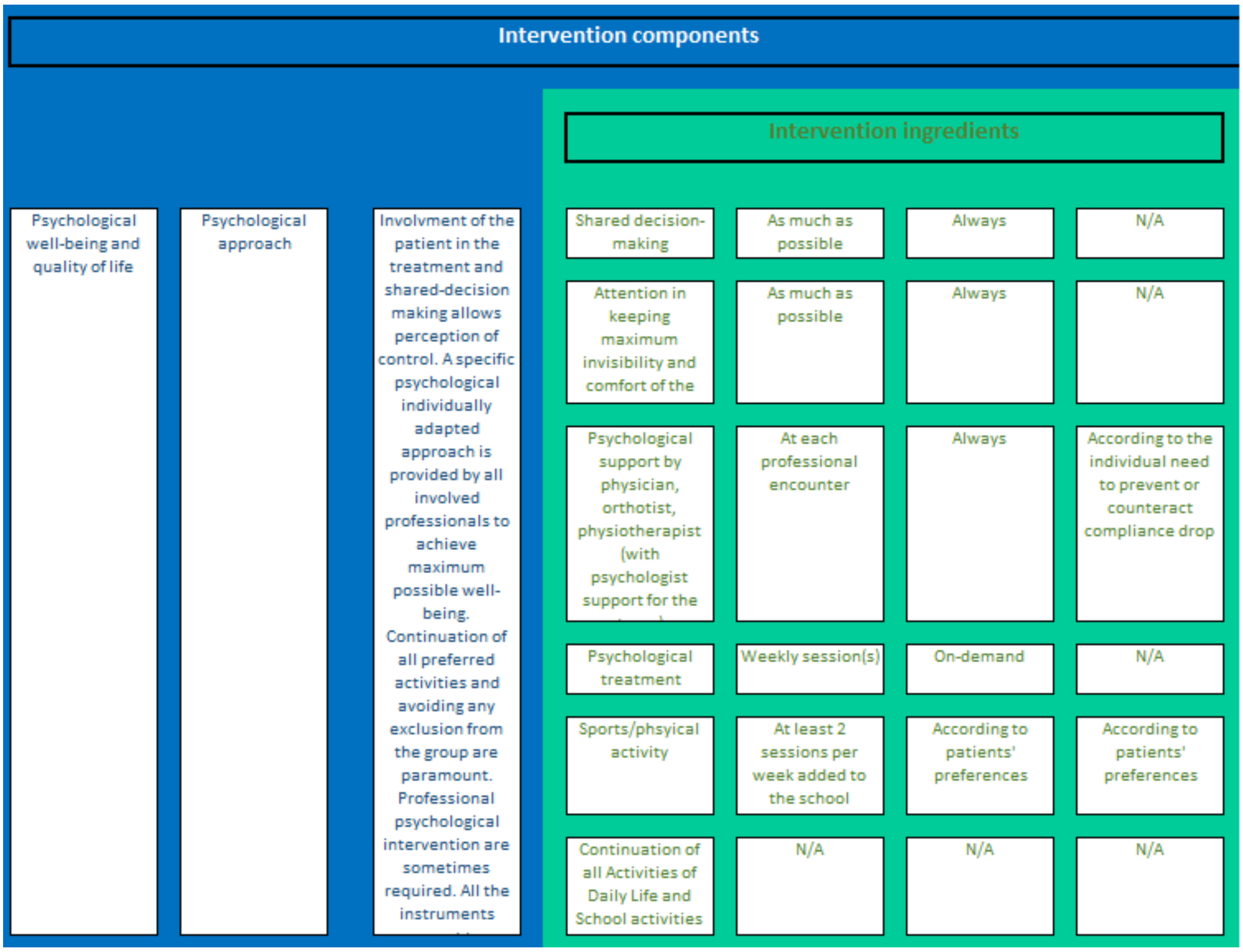

Figure 8 GUIDE-Rehab Programme 2 component 5 for bracing rehabilitation treatment

2) Overall description (PSSE): PSSE describes the exercises performed to treat idiopathic scoliosis patients. Specific exercises are combined with a cognitive-behavioural approach and a series of educational interventions and supports. Exercise treatment is the key to an effective rehabilitation approach for scoliosis. Rehabilitation focuses on the entire person. A good rehabilitation approach requires the means to compensate for, or prevent, if possible, psychological as well as physical and functional disabilities (transient in the case of bracing, definitive in the case of surgery). When other therapies are not yet considered, exercise treatment primarily aims to prevent scoliosis progression and should be applied whenever the risk of progression is significant. Exercises immediately follow observation alone and come before bracing. The aim is to avoid or at least postpone bracing and to arrive at the end of growth with a presumably stable curvature (as much as possible, far from 30°, so that a value between 20° and 25° can be acceptable). PSSE should never be proposed (in favour of bracing) when 30° curves have been attained unless the pubertal growth spurt is very far in the future and an important postural component is presumed, with the only aim of postponing (possibly avoiding) bracing. Moreover, exercises should be proposed when there are uncertainties regarding the application of a brace, even in curvatures exceeding 25°, and when stability is possible due to the absence of other progression factors and relatively advanced age. In such cases, it is important to make decisions together with the patient and their family. When a brace has already been prescribed, exercises are mandatory to avoid bracing-related side effects, improve its function, and maintain spine stability during the weaning period and after the brace is abandoned.

Intervention theory (PSSE): PSSE teaches Active Self-Correction through different movement and stabilisation exercises. The aim is to provide automatic counter-scoliosis postural support and movement, increasing neuromotor capacity and strengthening spinal stability through self-correction. A cognitive-behavioural approach helps us psychologically and allows us to achieve the best possible compliance. Sports activities potentiate the effect and have psychological value. Motivational tools are regularly developed and made available through a website and other educational materials; regular

motivational emails and phone communications, video consultations, or in-person meetings are also used as needed.

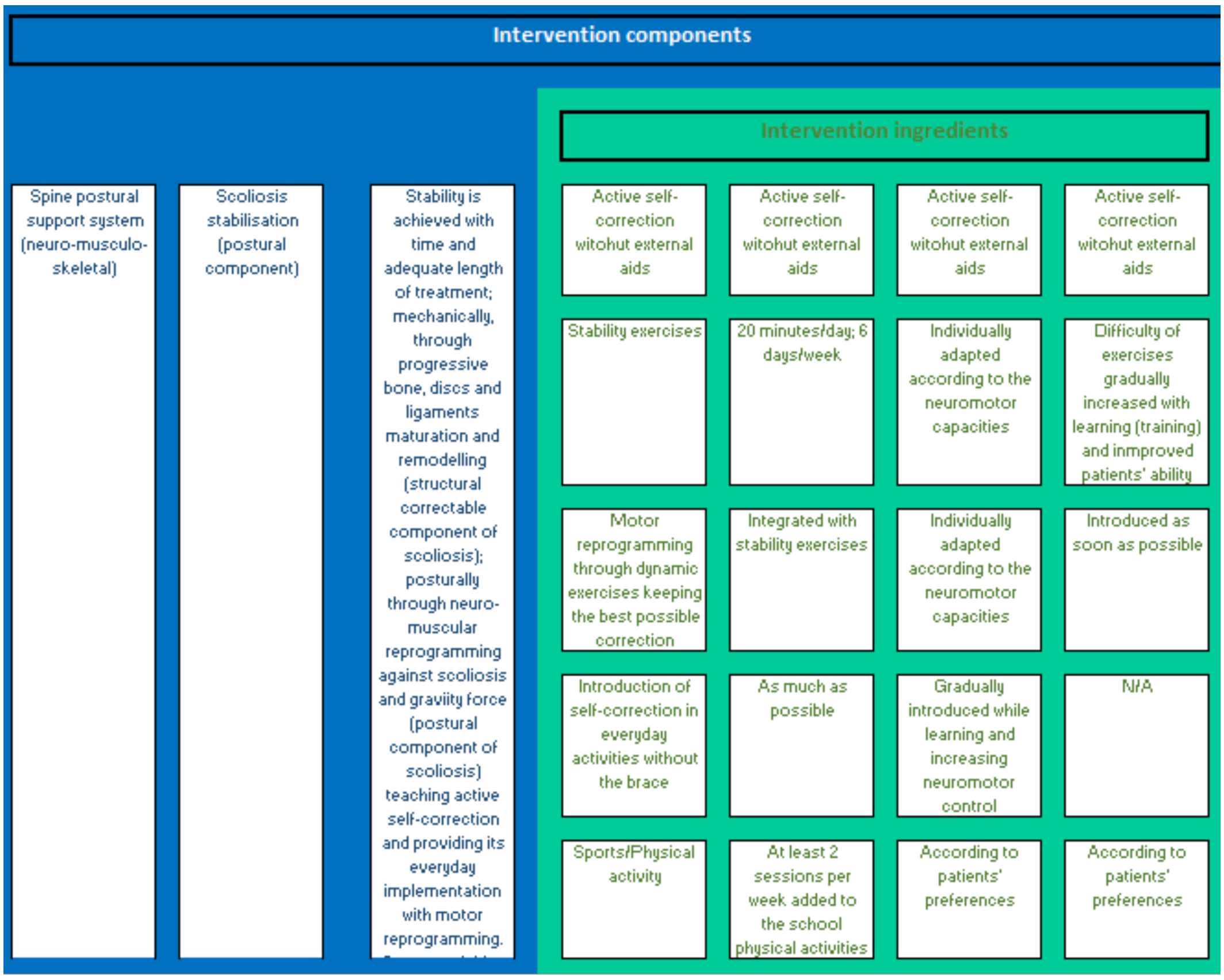

Figure 9 GUIDE-Rehab Programme 2 component 1 for PSSE rehabilitation treatment

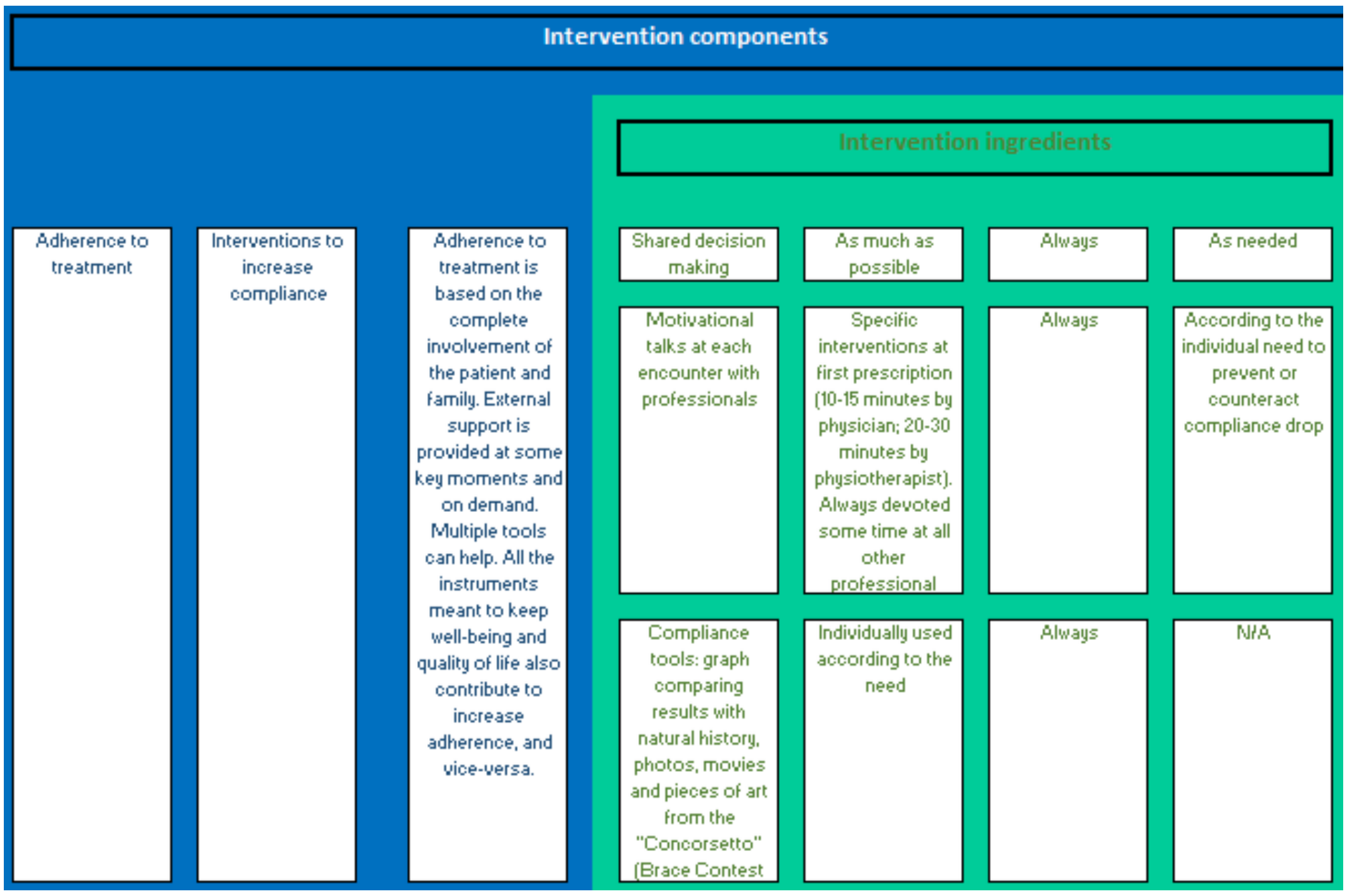

Figure 10 GUIDE-Rehab Programme 2 component 2 for PSSE rehabilitation treatment

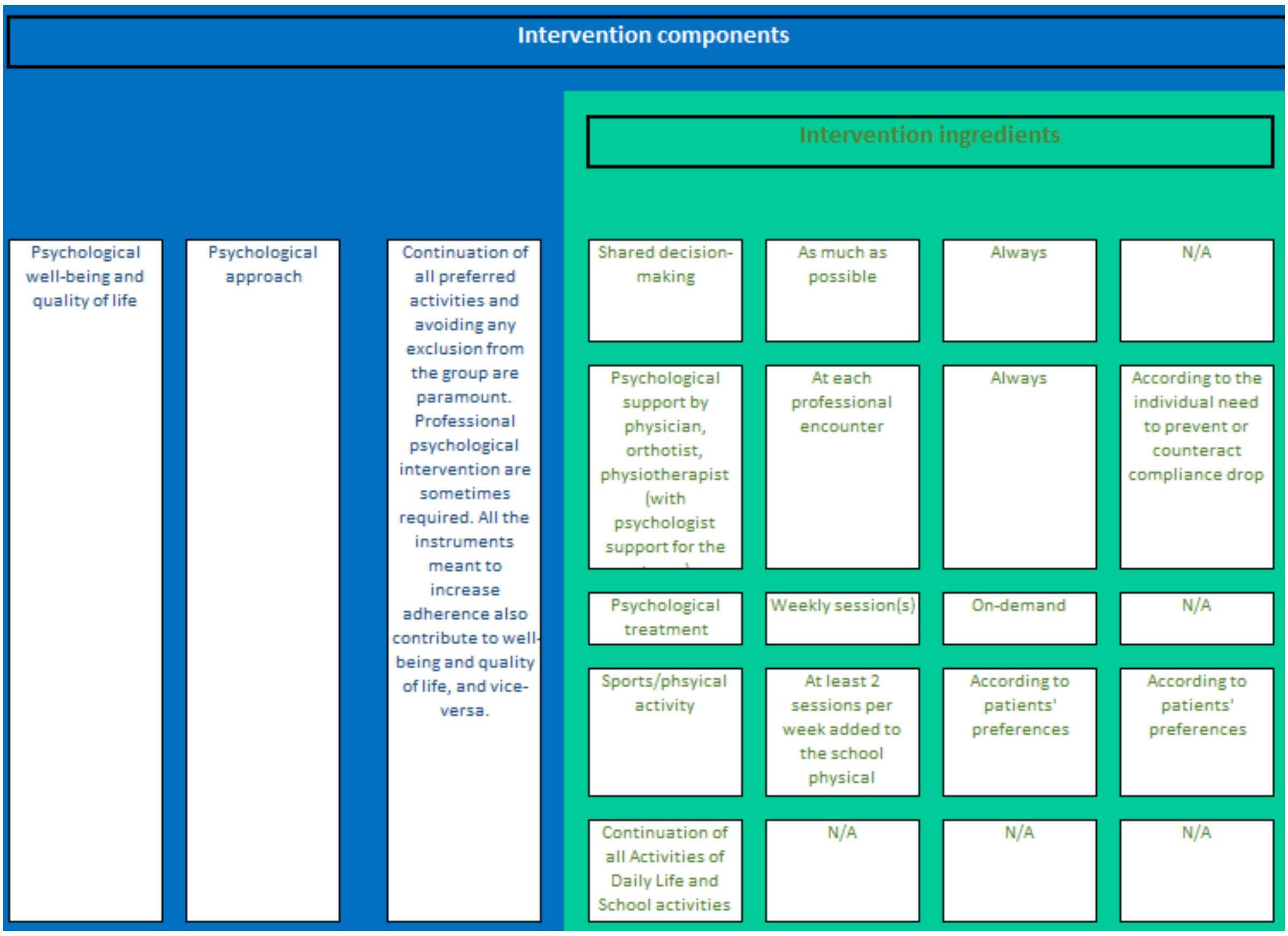

Figure 11 GUIDE-Rehab Programme 2 component 3 for PSSE rehabilitation treatment

Programme 3: Rehabilitation for people with intermittent claudication (Partner RAD)

Overall description: The principal goals of the interventions are the following: a) facilitating patients' attainment of desired functional activities and participation, thereby addressing individual needs, b) mitigating risk factors linked to atherosclerosis, c) empowering patients with progressive self-management skills in exercise training and adherence to an active lifestyle, d) enhancing patients' overall quality of life. During the initial 4 weeks, patients undergo an average of 3 sessions/week. Then the frequency is reduced to 2 sessions/week until the conclusion of the third month. From months 4 to 12, patients generally transition to a maintenance phase, with 1-2 monthly sessions. Regular quarterly follow-ups are advocated. 40 sessions are provided within the first year. A personalised education plan is developed based on the patient's information, advice, and guidance needs. The primary goal of exercise is to increase functional walking distance. The intensity and duration of training sessions should be gradually increased under a therapist's supervision, based on the patient's baseline walking distance. Patient preferences should be considered. Psychological support and peer interaction can help manage symptoms effectively. Behaviour-oriented rehabilitation principles can be beneficial, focusing on behaviour and situational awareness rather than underlying pathology. Therapists can apply motivational interviewing as a tool to promote behavioural change (encouraging physical activity, smoking cessation, adopting a healthy diet, and promoting medication adherence). Therapists may also collaborate with other healthcare professionals, such as dietitians and general practitioners, to effectively address patients' needs.

Intervention theory: treadmill training effectively increases walking distance and functional ability. For other treatment interventions, there is less evidence. However, through functional training, lifestyle interventions, and improvement of self-management, patients are expected to be able to manage their health independently and improve their quality of life. In general, conservative care is more cost-effective than surgical treatment.

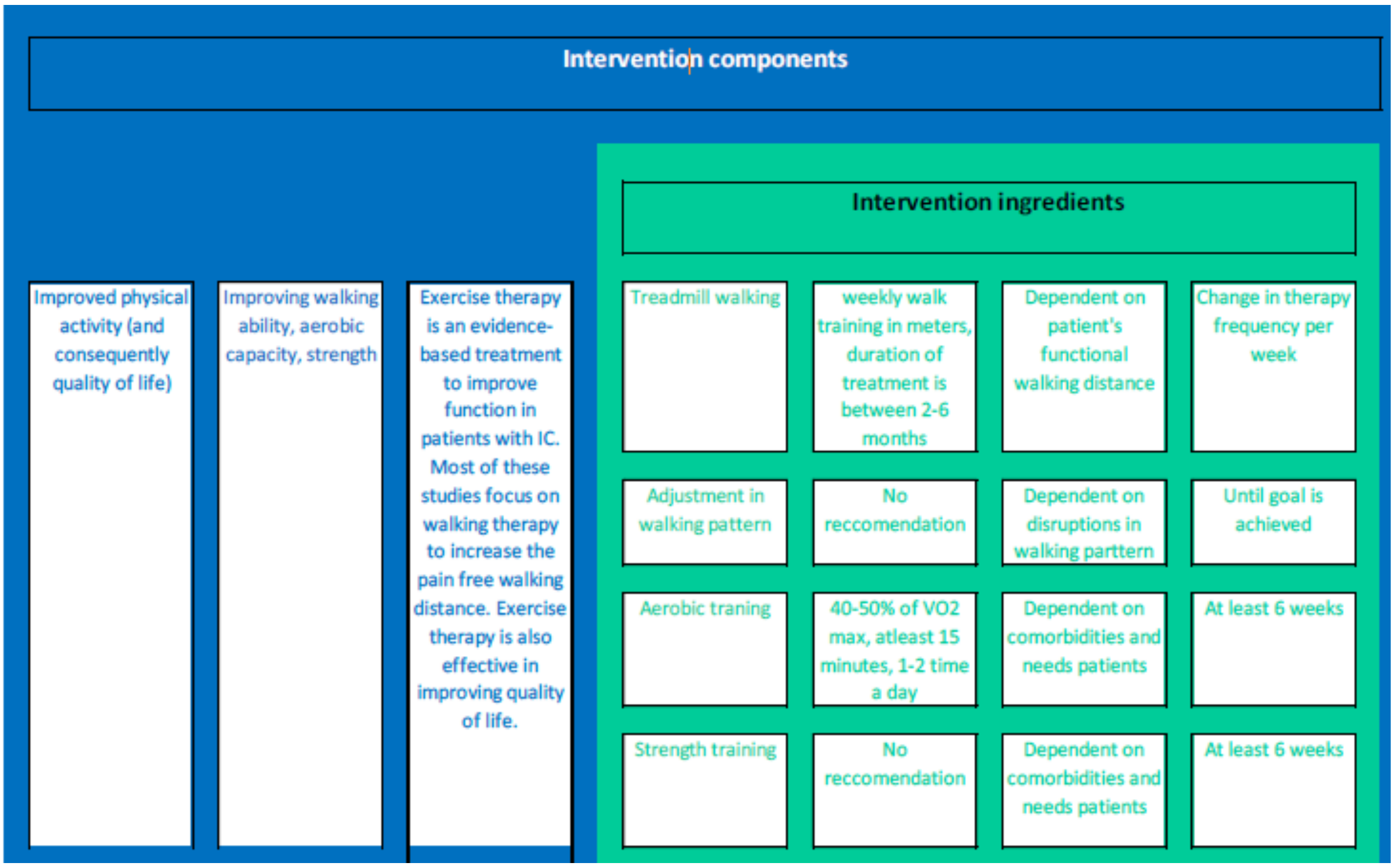

Figure 12 GUIDE-Rehab Programme 3 component 1

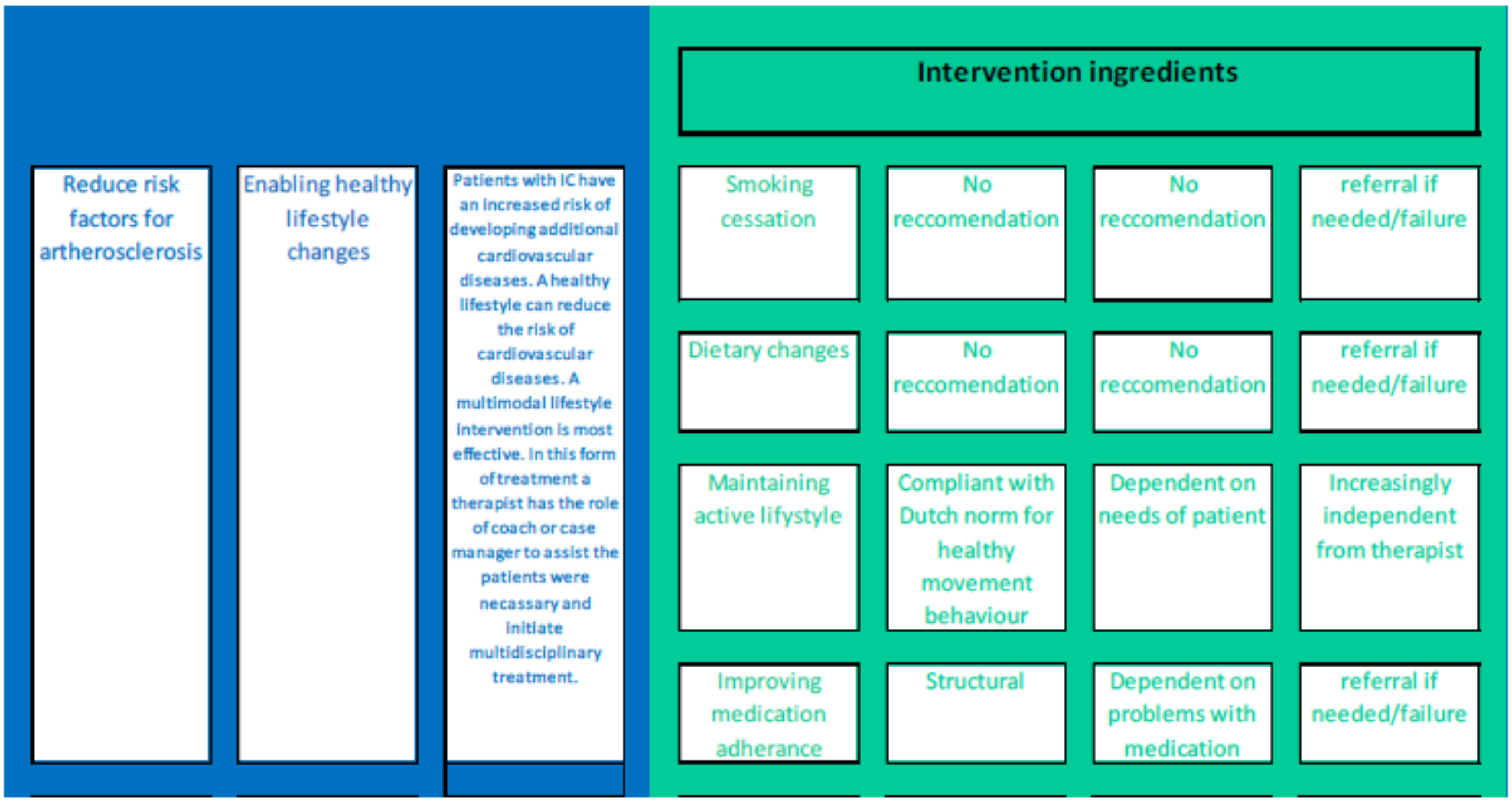

Figure 13 GUIDE-Rehab Programme 3 component 2

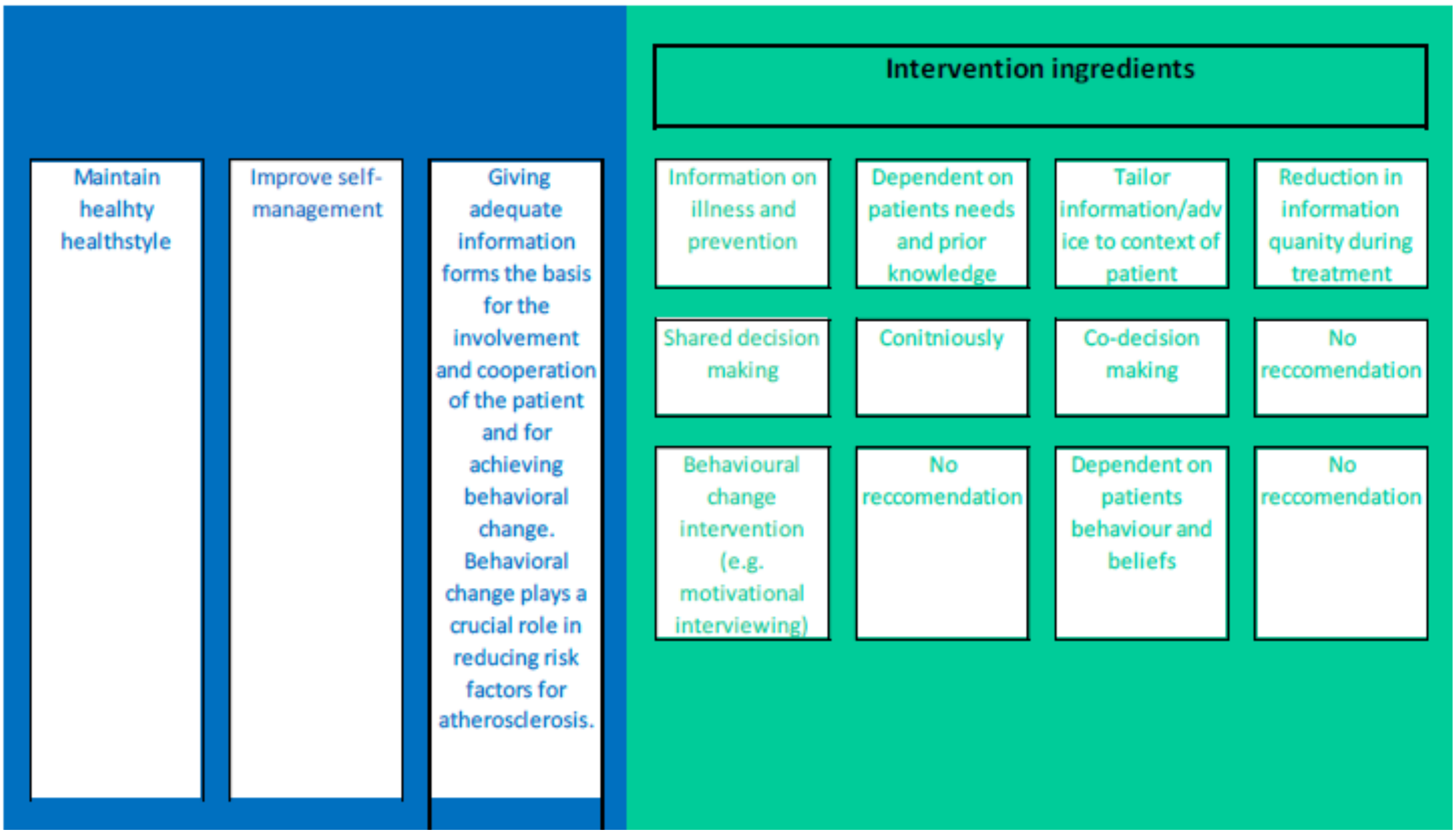

Figure 14 GUIDE-Rehab Programme 3 component 3

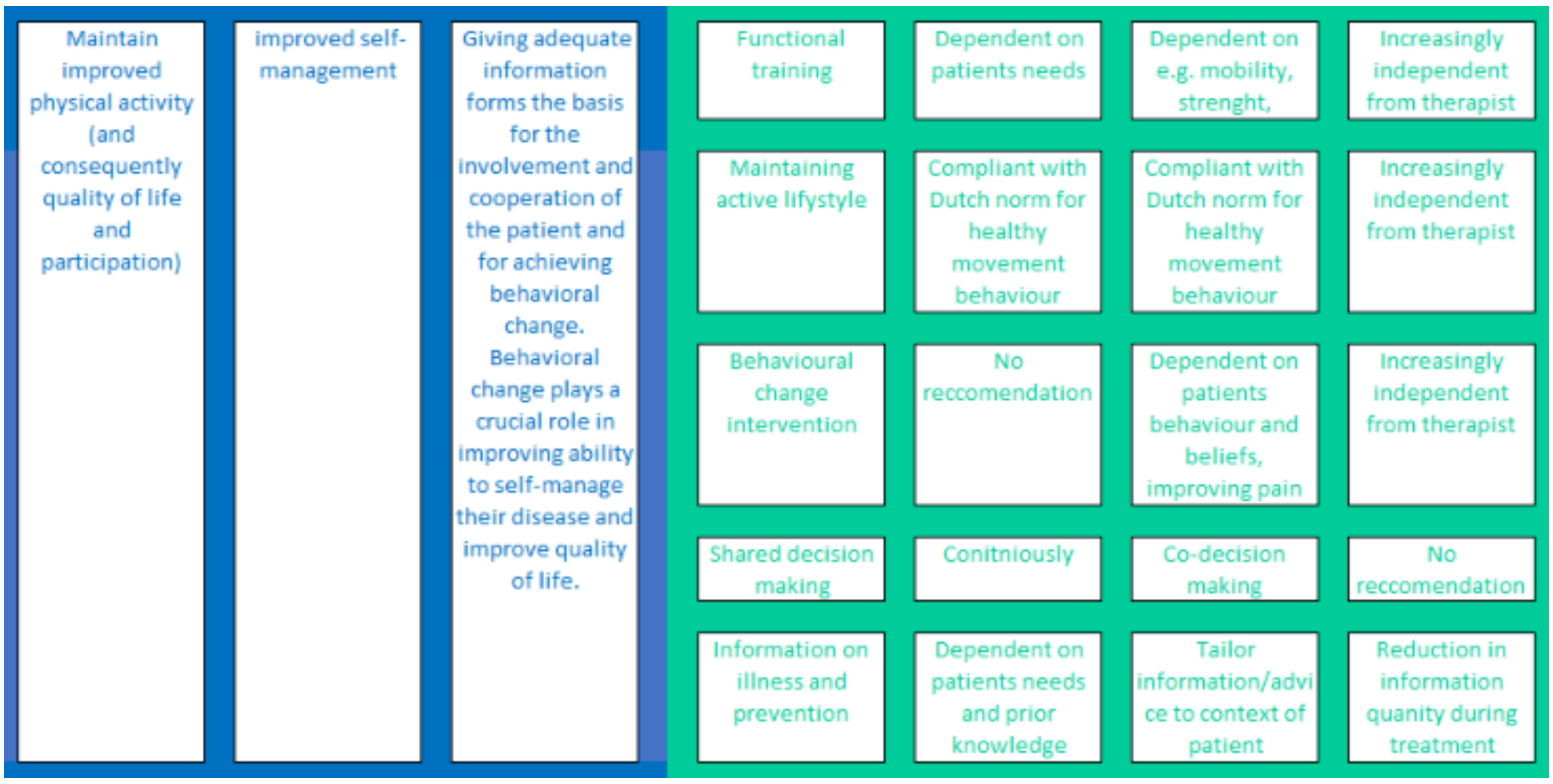

Figure 15 GUIDE-Rehab Programme 3 component 4

Programme 4: Rehabilitation for people with lower limb loss (Partner URI)

Overall description: After lower limb loss, all patients are referred to the outpatient clinic and admitted for comprehensive rehabilitation. At admission, patients undergo extensive testing in the first three days. The results are discussed at the first team meeting to define further interventions. Multimodal training is performed according to internationally published guidelines (1). The aim of the intervention is to increase cardiorespiratory fitness, improve ADL, and enhance gait endurance and dynamic balance. Important aspects of multimodal rehabilitation also relate to falls and wounds prevention.
Intervention theory: Within the multimodal training, it is expected that education on proper execution of ADL, removal of barriers in the home environment, balance training, and use of appropriate walking aids significantly reduce the number of falls.

1. Geertzen J, van der Linde H, Rosenbrand K, Conradi M, Deckers J, Koning J, et al. Dutch evidence-based guidelines for amputation and prosthetics of the lower extremity: Rehabilitation process and prosthetics. Part 2. Prosthet Orthot Int 2015; 39(5): 361–71. DOI: 10.1177/0309364614542725

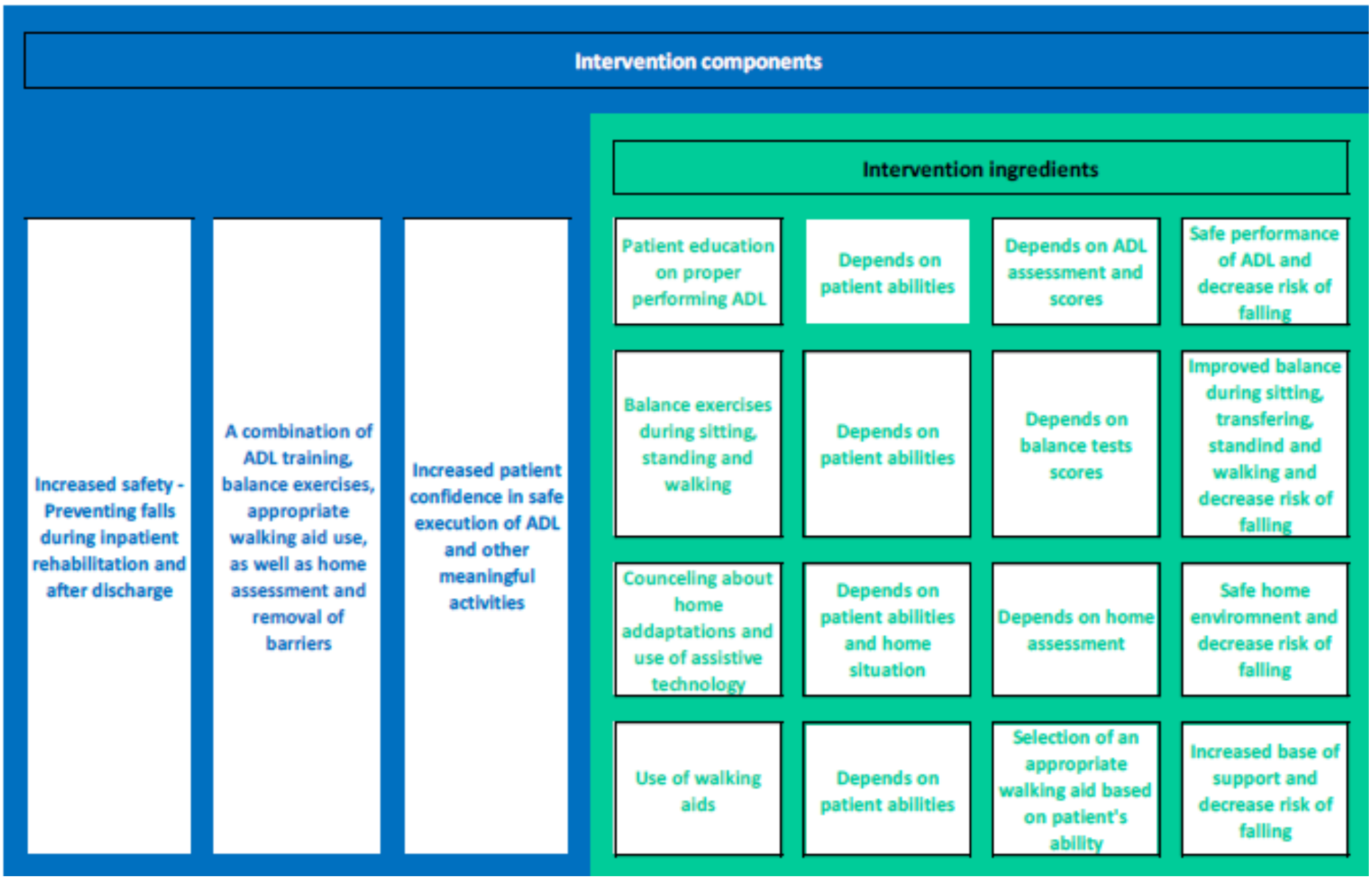

Figure 16 GUIDE-Rehab Programme 4 component 1

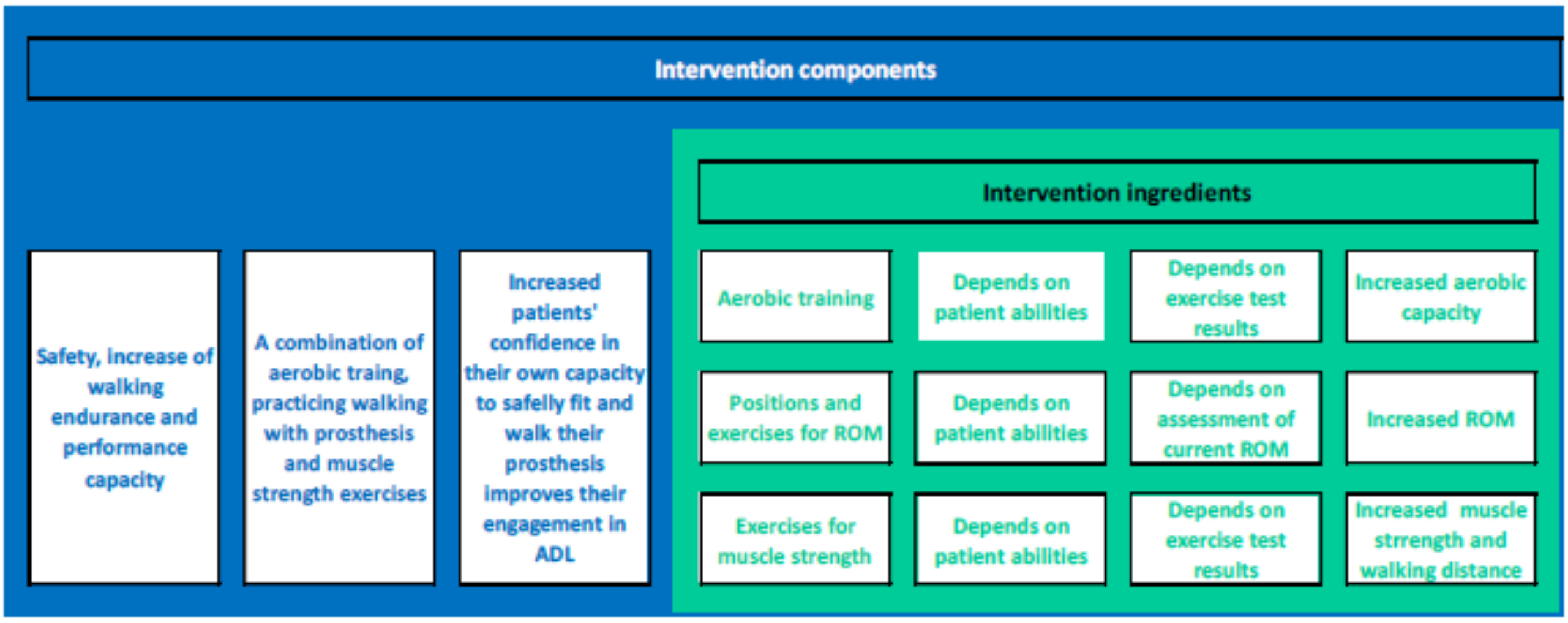

Figure 17 GUIDE-Rehab Programme 4 component 2

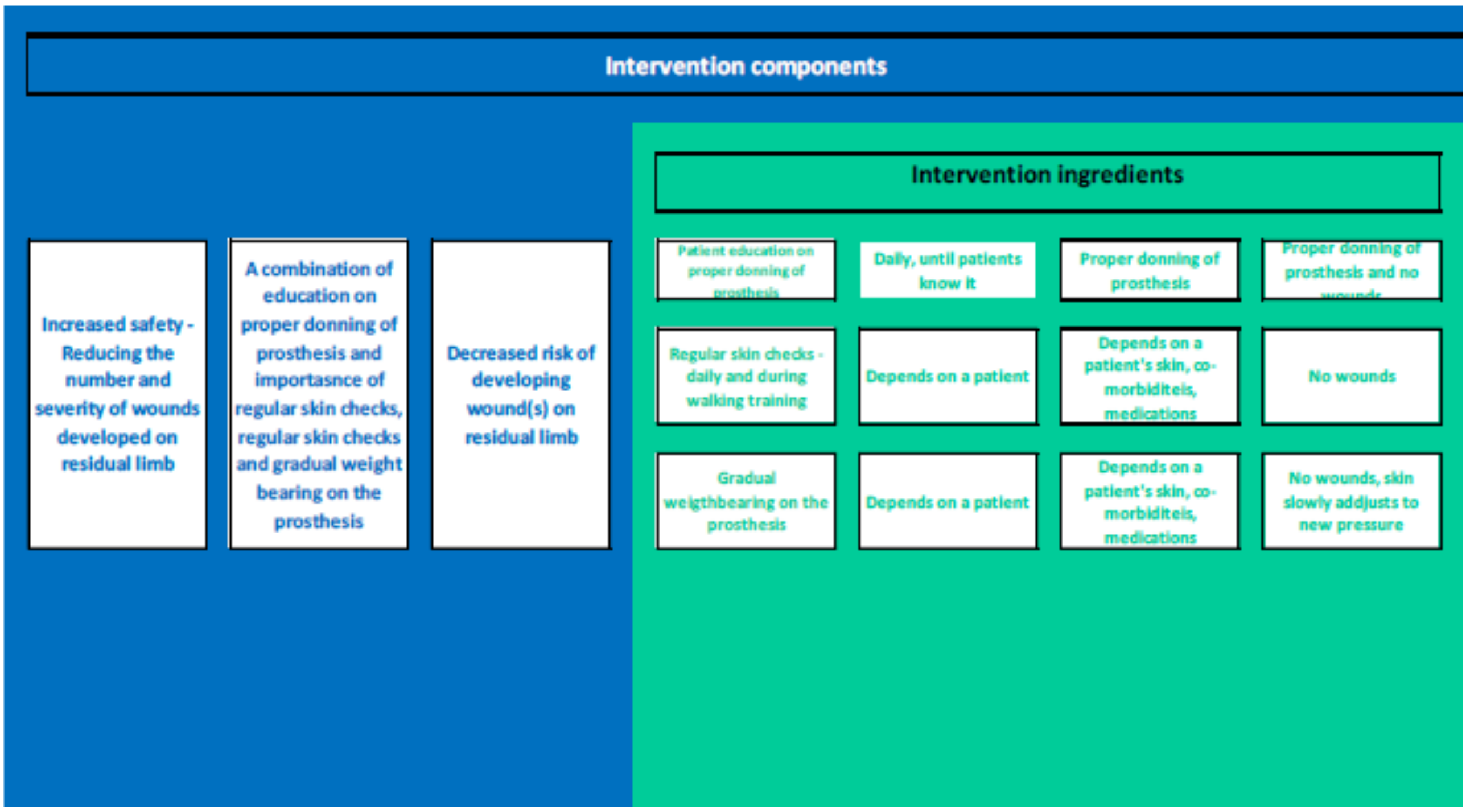

Figure 18 GUIDE-Rehab Programme 4 component 3

Programme 5: Rehabilitation for people with Parkinson's disease and Parkinsonism (Partner UNIVPM)

Overall description: People with Parkinson's disease (PD) or Parkinsonism may benefit from rehabilitation approaches in any disease stage. In the early to intermediate disease stage, *high-intensity multimodal training* is prescribed to increase cardiorespiratory fitness, overcome psychological barriers to regular physical activity, and promote independence in ADL by targeting functions that may be impaired (speech, hand dexterity, gait, and balance). High-intensity training consists of at least 1800 minutes multimodal practice over a 2-3 months period, to be repeated twice per year to achieve long-lasting benefits. Occupational therapy targets independence in ADL; physical therapy targets gait and balance by teaching patients the correct use of cueing strategies; aerobic training enhances cardiovascular fitness and endurance, whereas action observation with the intention of gesture imitation improves arm movement speed- People with PD experiencing motor and non-motor fluctuations, with or without disabling dyskinesia as side effects of long-term levodopa treatment, are referred for functional neurosurgery for deep brain stimulation, provided they meet specific eligibility criteria (i.e., no levodopa refractory motor and non-motor symptoms, no severe comorbidities, no previous brain surgery or brain damage other than neurodegeneration). Finally, patients in any disease stage are offered *telerehabilitation* protocols tailored to their disability profile. Eligibility criteria for this approach include patients' motivation, retained ability to interact with the online platform that provides a choice of video-recorded exercises to be observed and imitated, caregiver collaboration, and a stable internet connection.

Intervention theory (High-intensity multimodal training): aims to increase cardiorespiratory fitness, overcome psychological restraints preventing people from engaging in regular physical activity, and promote independence in ADL by targeting different functions that may be impaired in people with PD (namely, speech, hand dexterity, balance).

Intervention theory (functional neurosurgery for deep brain stimulation): Deep brain stimulation of subthalamic nuclei is an established effective approach to the symptomatic treatment of cardinal motor features in people with PD. Specifically, the activation of electromagnetic fields within the subthalamus produces a modulation of the cortical-thalamic-striatal pathway, able to achieve the

same improvement in the cardinal motor features (i.e., rest tremor, rigidity, bradykinesia) as the optimal levodopa dose.

Intervention theory (*telerehabilitation):* Telerehabilitation (TR) allows training for longer periods in real-world scenarios. TR can be highly motivating and challenging, increasing treatment adherence. Access to specialised care can be difficult, and TR and telemonitoring are viable options for managing patients who require an ongoing, long-term approach. The economic and human burden of care of PD is high, and TR is proposed as a tool to ensure personalised and equally distributed continuous training.

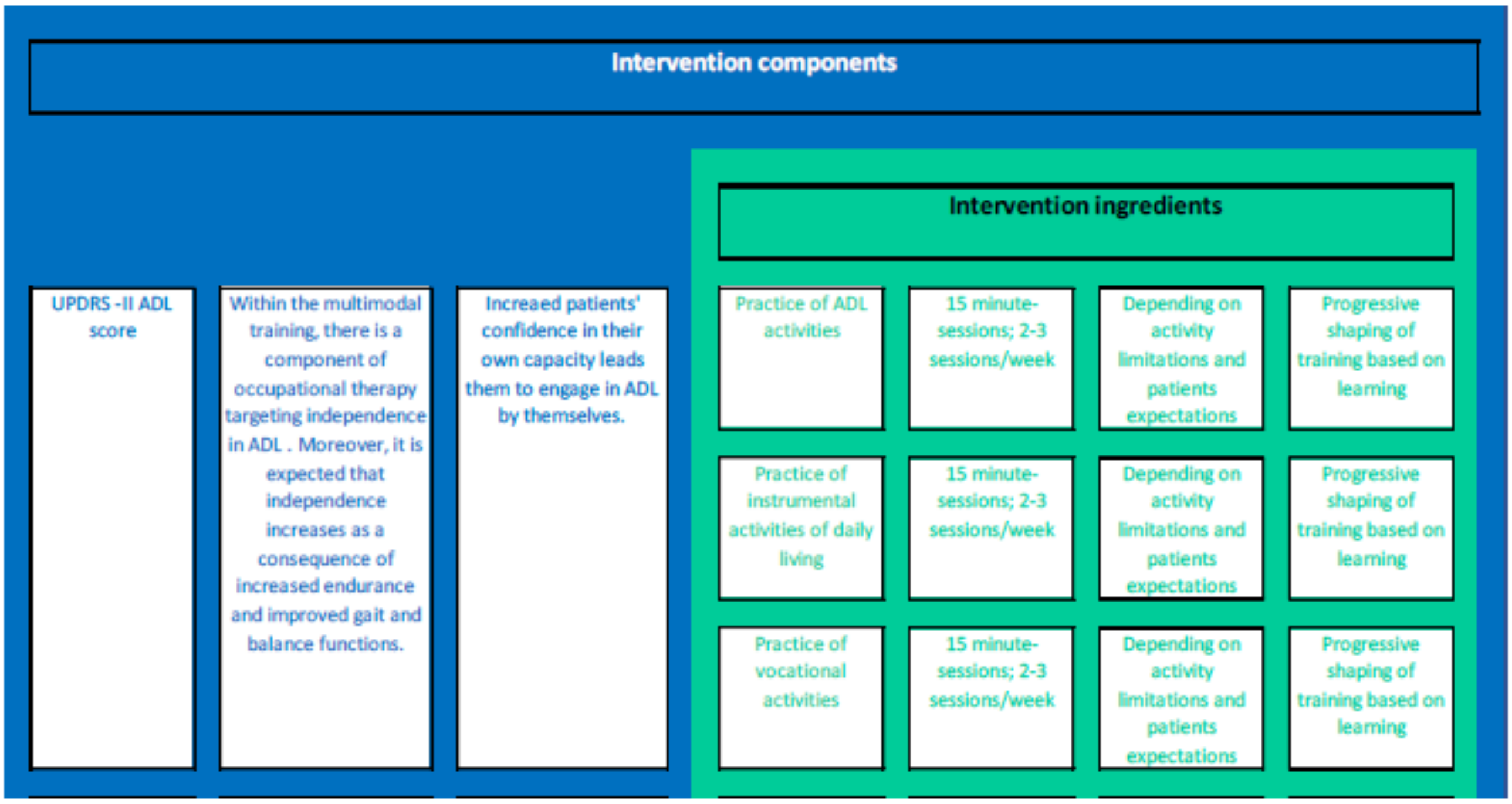

Figure 19 GUIDE-Rehab Programme 5 component 1 for High-intensity multimodal training (HIT)

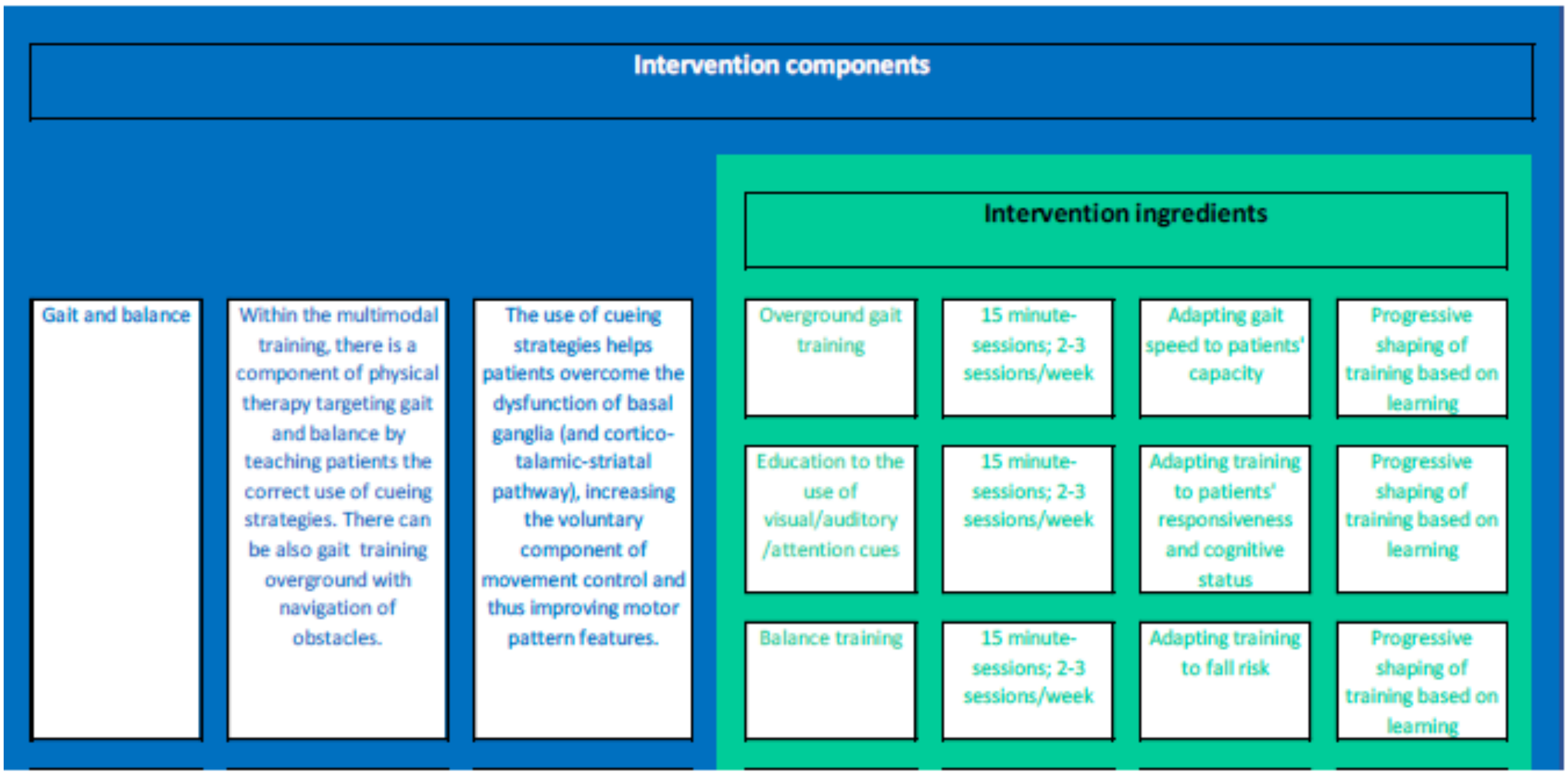

Figure 20 GUIDE-Rehab Programme 5 component 2 for High-intensity multimodal training (HIT)

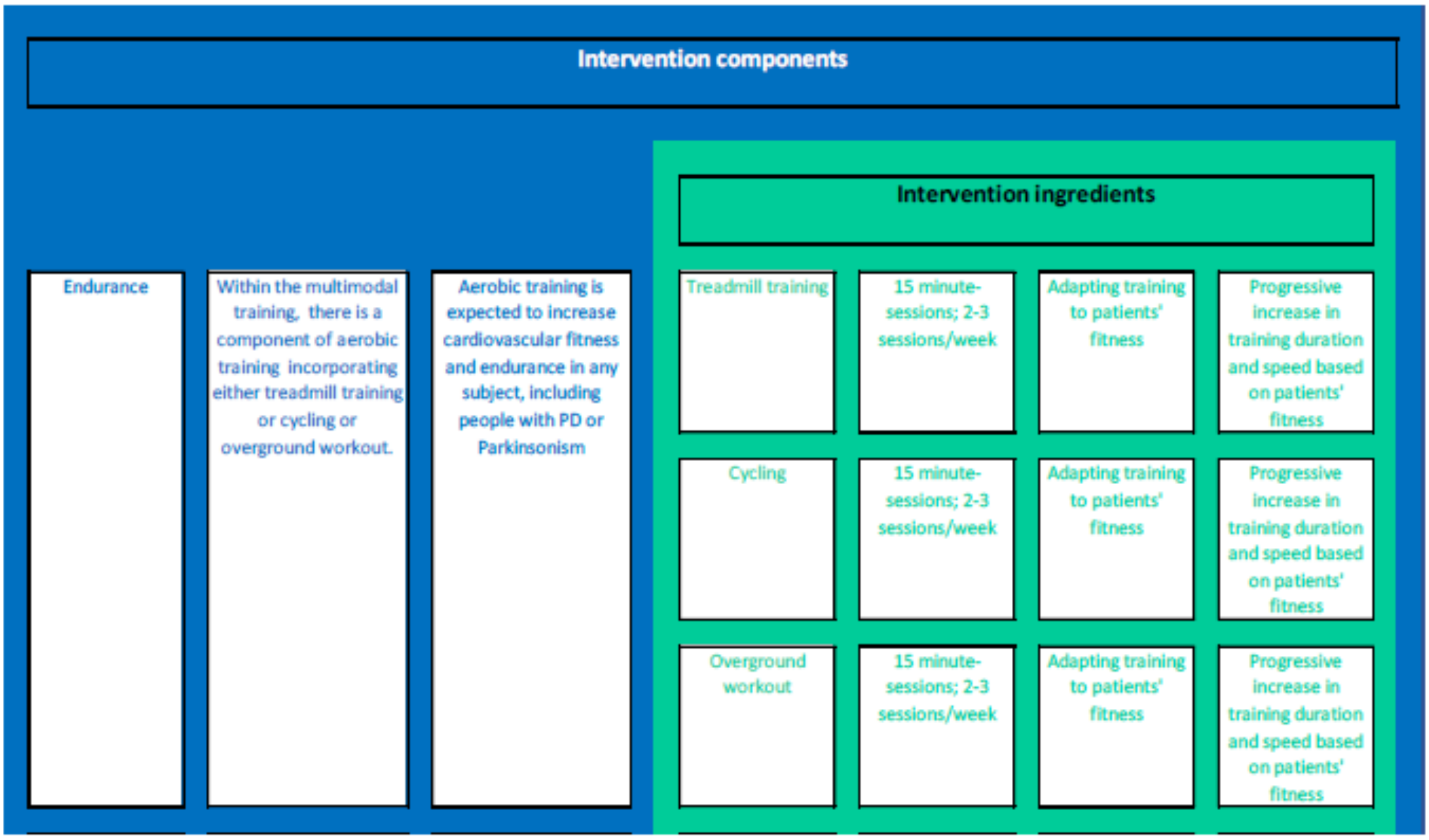

Figure 21 GUIDE-Rehab Programme 5 component 3 for High-intensity multimodal training (HIT)

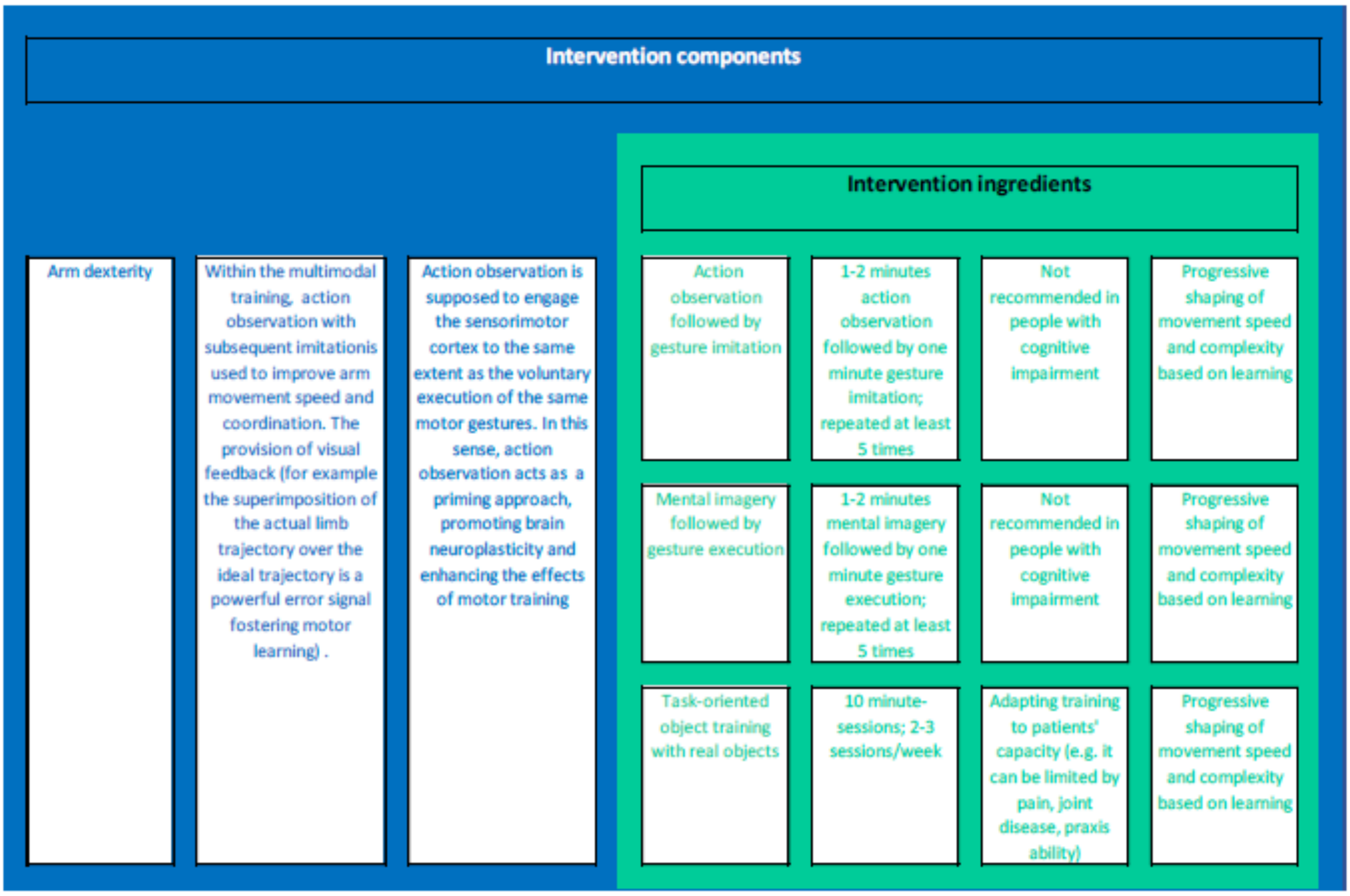

Figure 22 GUIDE-Rehab Programme 5 component 4 for High-intensity multimodal training (HIT)

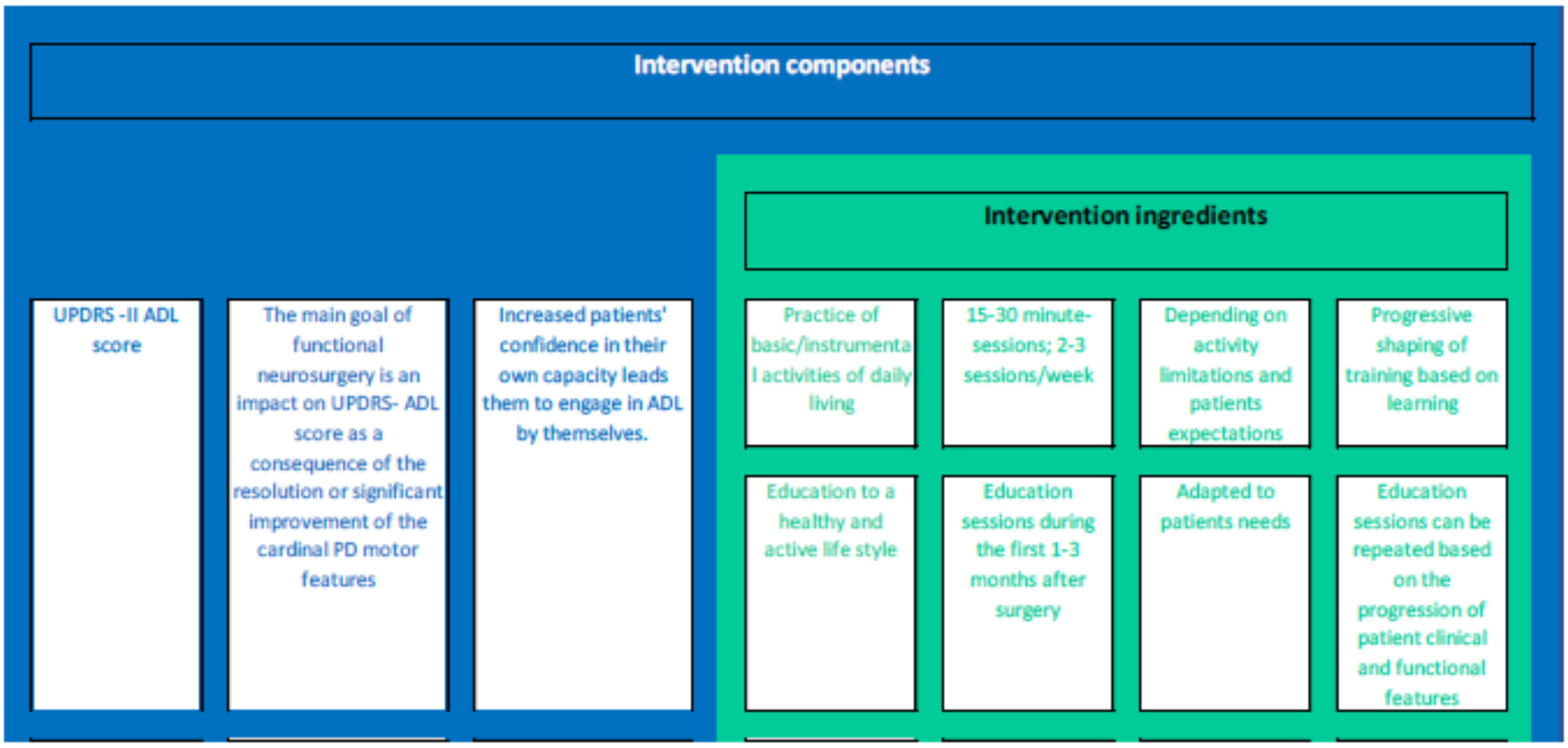

Figure 23 GUIDE-Rehab Programme 5 component 1 for functional neurosurgery for deep brain stimulation

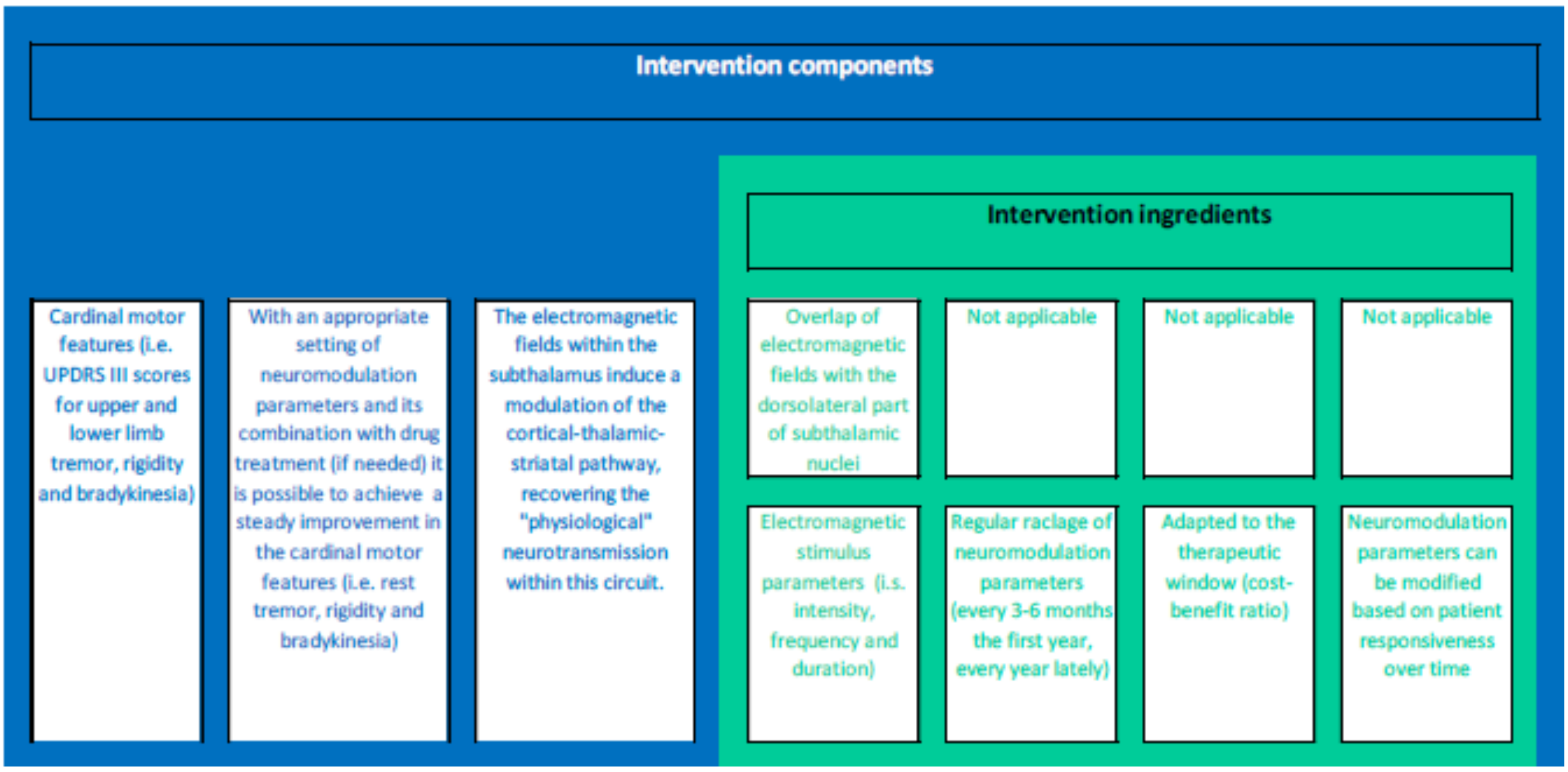

Figure 24 GUIDE-Rehab Programme 5 component 2 for functional neurosurgery for deep brain stimulation

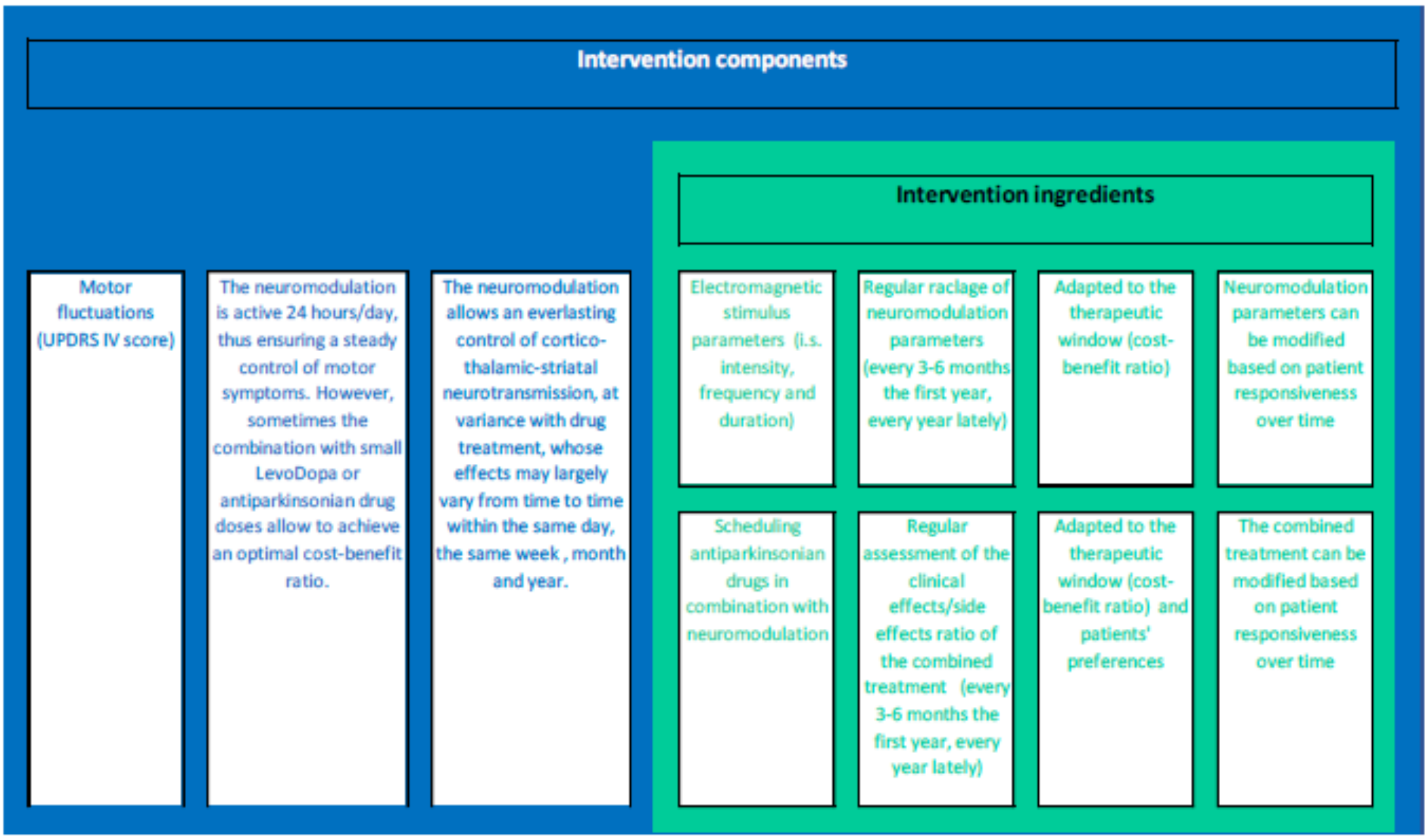

Figure 25 GUIDE-Rehab Programme 5 component 3 for functional neurosurgery for deep brain stimulation

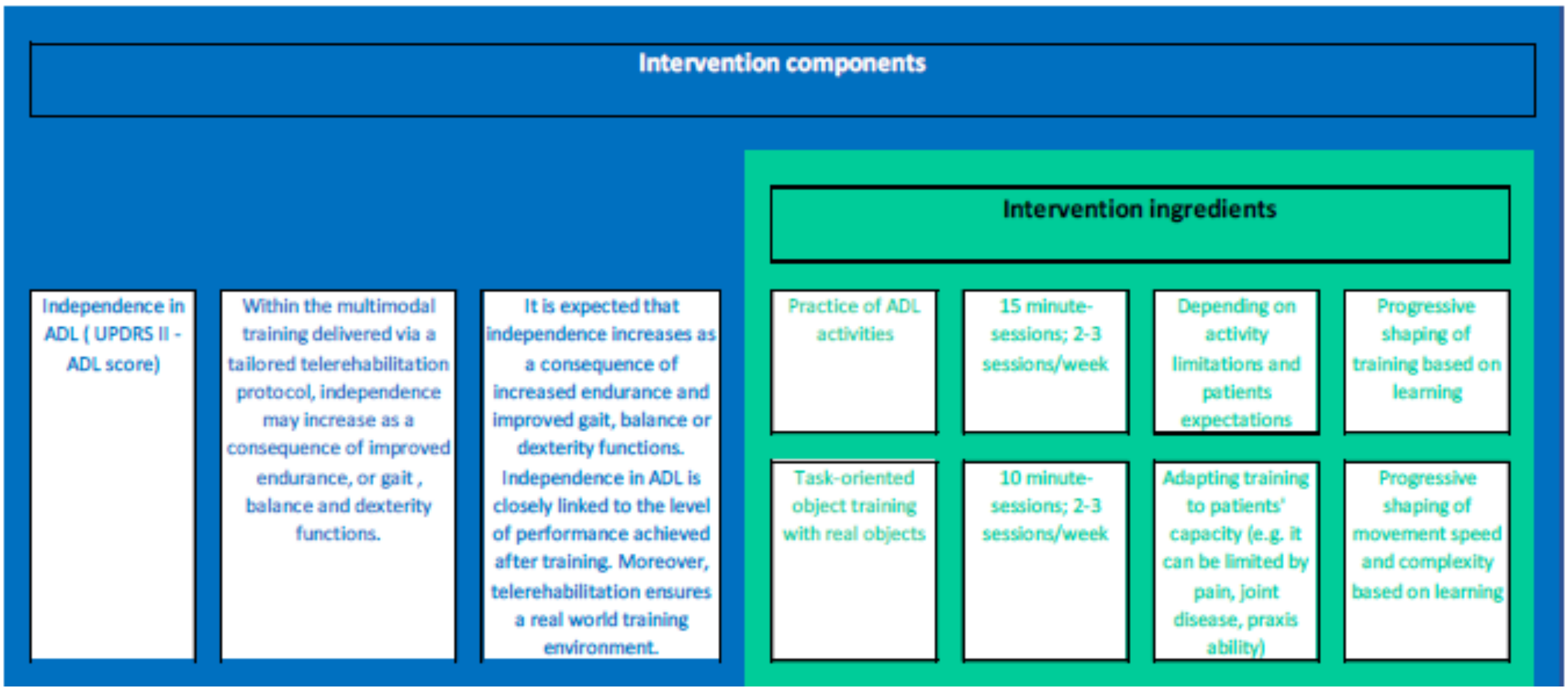

Figure 26 GUIDE-Rehab Programme 5 component 1 for telerehabilitation

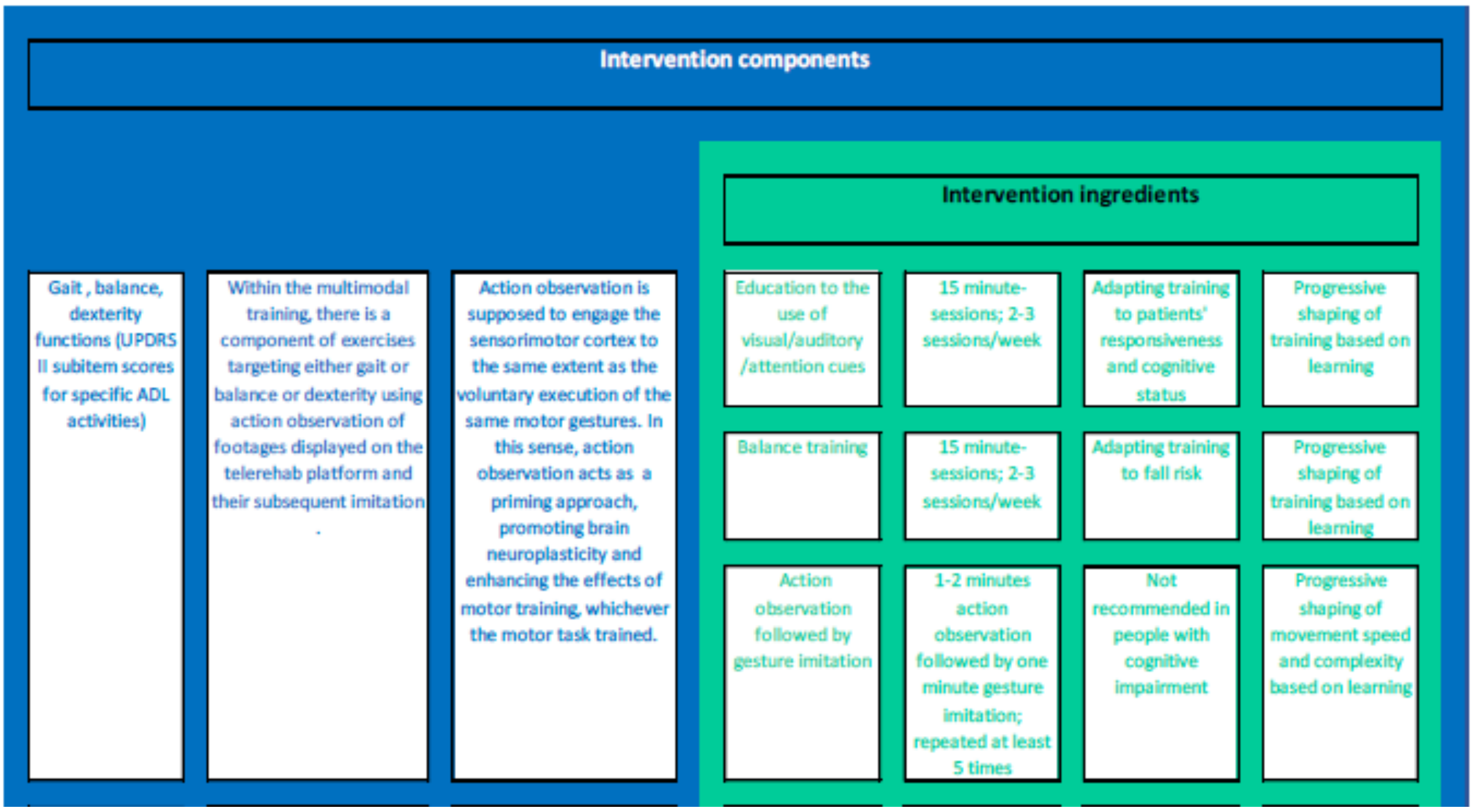

Figure 27 GUIDE-Rehab Programme 5 component 2 for telerehabilitation

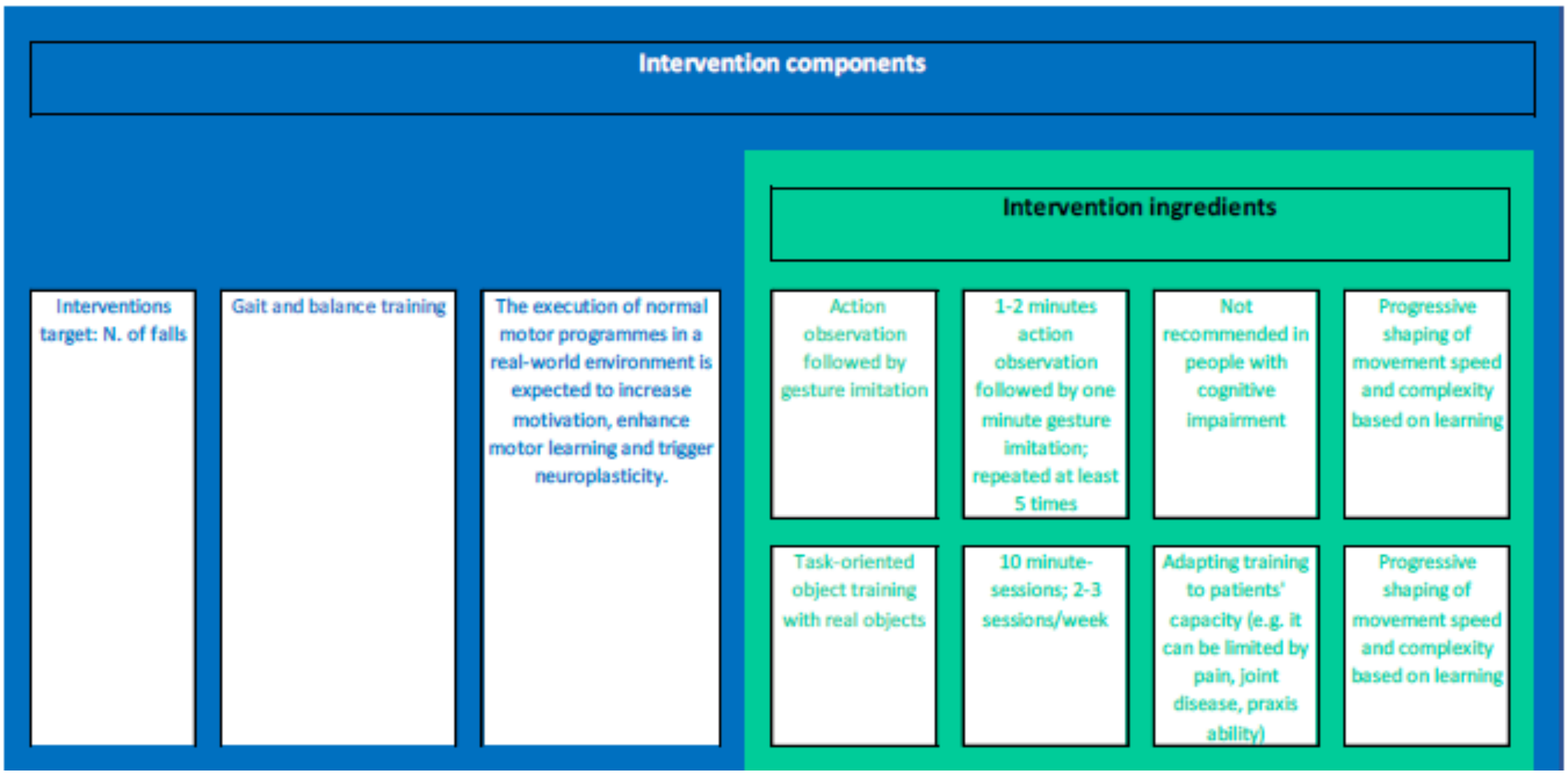

Figure 28 GUIDE-Rehab Programme 5 component 3 for telerehabilitation

Programme 6: Rehabilitation for people after hip and knee replacement for osteoarthritis (Partner OGSA)

Overall description: Compatible with the absence of intraoperative complications, the patient's safety and drive, the patient is encouraged to get out of bed as soon as possible, even on the evening of the same day of surgery. However, most of the time, this happens the next day. This is the only part of the rehabilitation program which is performed in the surgical ward. Joint recovery and muscle strengthening exercises are prescribed. The patient is trained to climb stair, which is a key step for discharge. Continuous passive motion is performed one hour a day, gradually improving the range of motion without pain. Depending on the specific surgical technique, the patient is trained to avoid risky postures and movement until they learn. Postural step and transfer training are also performed continuously,

compatible with the patient's ability. Recovery in ADL, with the main goal of making the patient autonomous in the ordinary actions they need to perform on their own outside the hospital. Transfers and climbing stairs training also fall into this category, as long as they are needed to make patients as autonomous as possible. Functional recovery to move independently is the priority. Eating, bathing, dressing, and toileting need training, but patients can be discharged from the hospital before they reach autonomy in these activities. Clinical stabilisation, with the main goals of controlling pain after surgery, restoring safe iron levels and preventing deep venous thromboembolism.

Intervention theory: Pharmacological pain treatment, Deep Venous Thrombosis prevention by means of pharmacological and mechanical means are prescribed. Exercises (joint and muscle strength recovery, postural steps and transfers training, climbing stairs, avoiding risky postures and movements, ADL) are prescribed to recover the maximum independence before discharge and achieve global and segmental functional recovery.

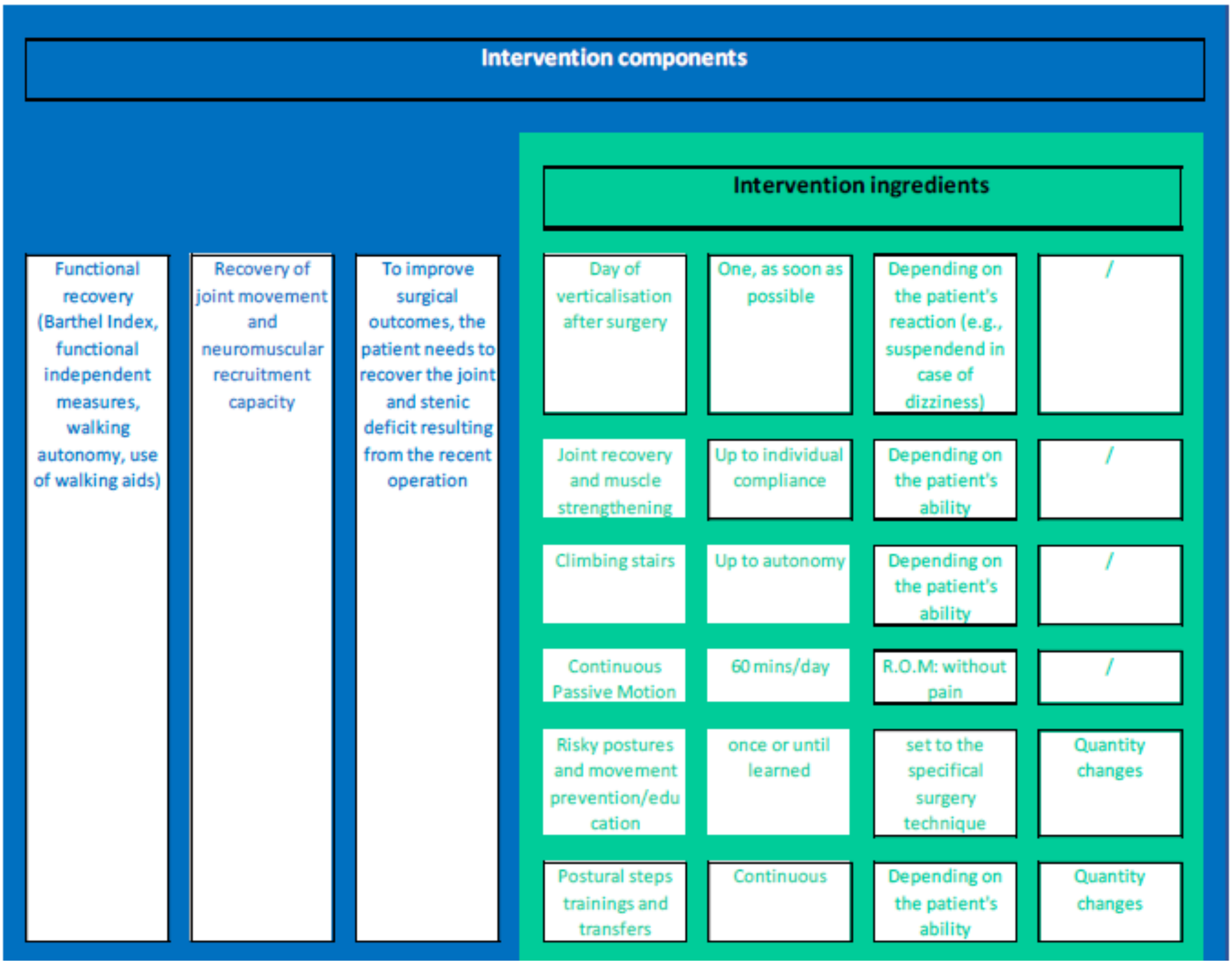

Figure 29 GUIDE-Rehab Programme 6 component 1 for inpatient rehabilitation after elective hip or knee total replacement

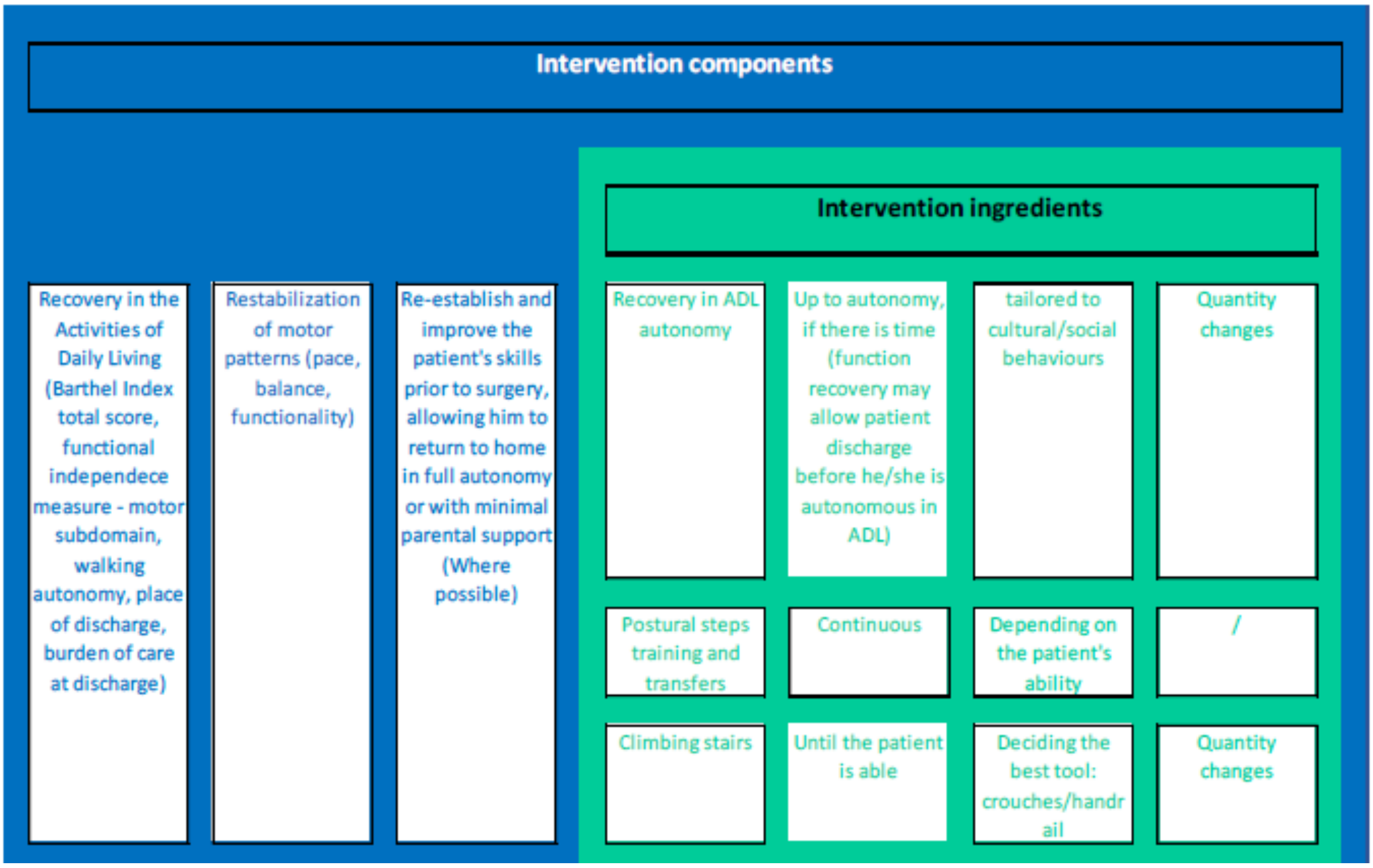

Figure 30 GUIDE-Rehab Programme 6 component 2 for inpatient rehabilitation after elective hip or knee total replacement

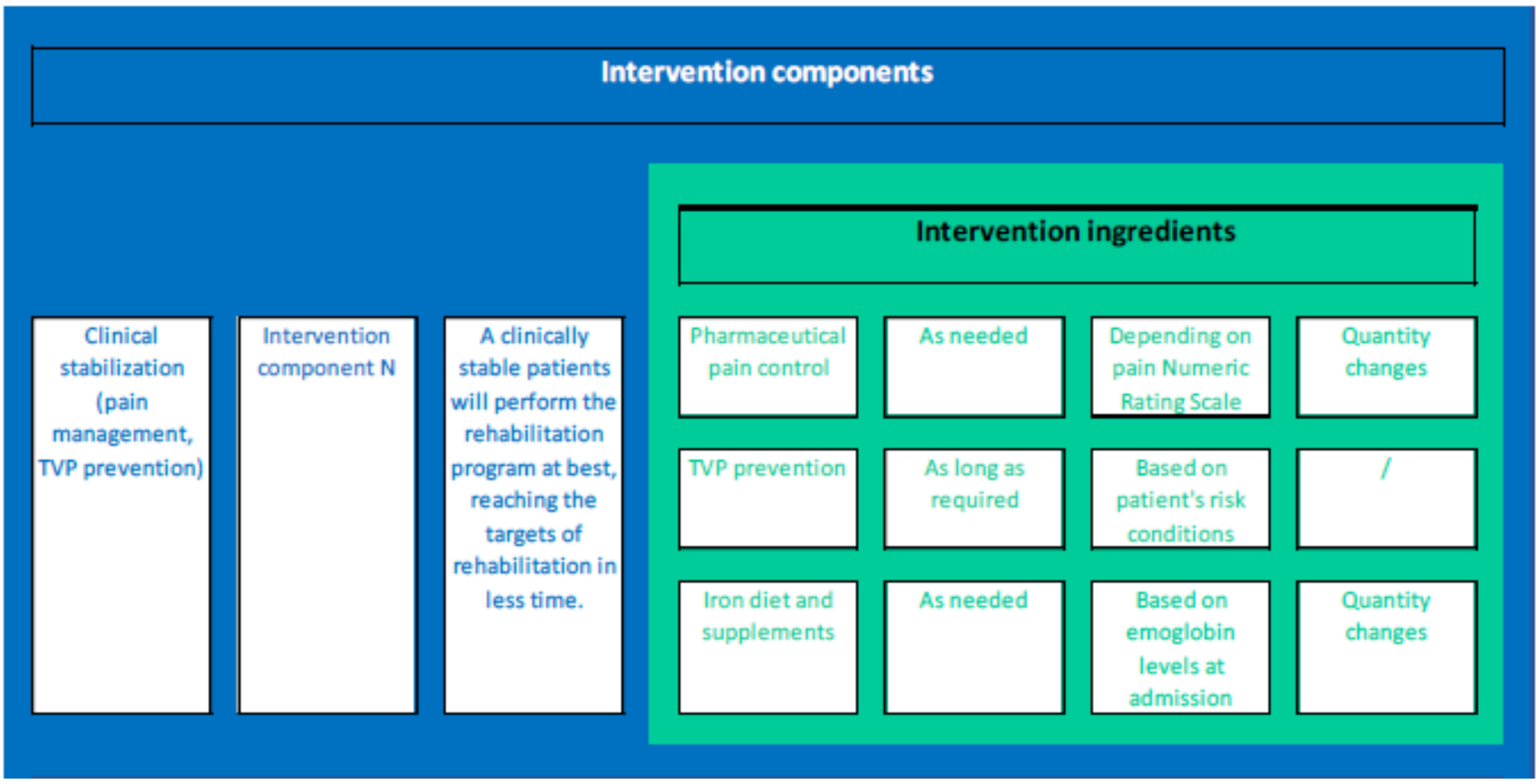

Figure 31 GUIDE-Rehab Programme 6 component 3 for inpatient rehabilitation after elective hip or knee total replacement

Programme 7: Rehabilitation for people with temporomandibular joint disorders (Partner UNIMIL

Overall description: The rehabilitation process for temporomandibular joint disorders (TMD) encompasses a multifaceted approach to address the underlying causes and alleviate symptoms. This process typically involves a combination of therapeutic interventions, lifestyle modifications, and patient education. The rehabilitation process begins with a thorough evaluation of the patient's medical history, symptoms, and physical examination findings. Diagnostic criteria stored within the database aid clinicians in accurately identifying TMD subtypes and formulating tailored treatment plans.

Treatment strategies encompass a range of modalities, including physical therapy, pharmacotherapy, occlusal splint therapy, and behavioural interventions. The database provides guidance on selecting appropriate therapies based on the patient's specific presentation and individual needs. Central to the rehabilitation process is empowering patients with the knowledge and skills to participate in their care actively. Clinicians utilise the database to access educational resources and provide guidance on self-management techniques, such as stress management and jaw exercises. Long-Term Monitoring and Follow-Up: Ongoing monitoring is essential to track treatment progress, adjust interventions as needed, and prevent disease recurrence. The database facilitates longitudinal data collection, enabling clinicians to monitor outcomes over time and refine treatment approaches based on real-world effectiveness.

Intervention theory (Reversible Therapy): non-invasive or conservative treatments aim at improving symptoms and functional limitations while minimising the need for invasive procedures. They focus on Self-Management; Physical therapy (exercises, stretches, and manual techniques designed to improve jaw mobility, reduce muscle tension, and enhance TMJ function); Occlusal splints (Custom-made oral appliances worn over the teeth to redistribute forces, reduce clenching or grinding, and provide temporary relief from TMJ pain); Pharmacotherapy (nonsteroidal anti-inflammatory drugs, muscle relaxants, or tricyclic antidepressants may be prescribed to manage pain, inflammation, or associated conditions like bruxism or anxiety); Behavioural therapy (stress management, relaxation training, and cognitive-behavioural therapy).

Intervention theory (Irreversible Therapy Rehab): invasive or surgical interventions that result in permanent alterations to the TMJ or surrounding structures. These are typically considered when conservative treatments fail to provide adequate relief or when structural abnormalities or pathology require more aggressive management. Examples of Irreversible therapies are: TMD Arthrocentesis; Arthroscopy; Open-joint surgery; Joint replacement. Irreversible therapy aims to address structural or pathological changes within the TMJ and may provide long-term relief for individuals with refractory or severe symptoms. However, these interventions carry greater risks and may involve longer recovery than reversible therapies, highlighting the importance of careful consideration and individualised treatment planning.

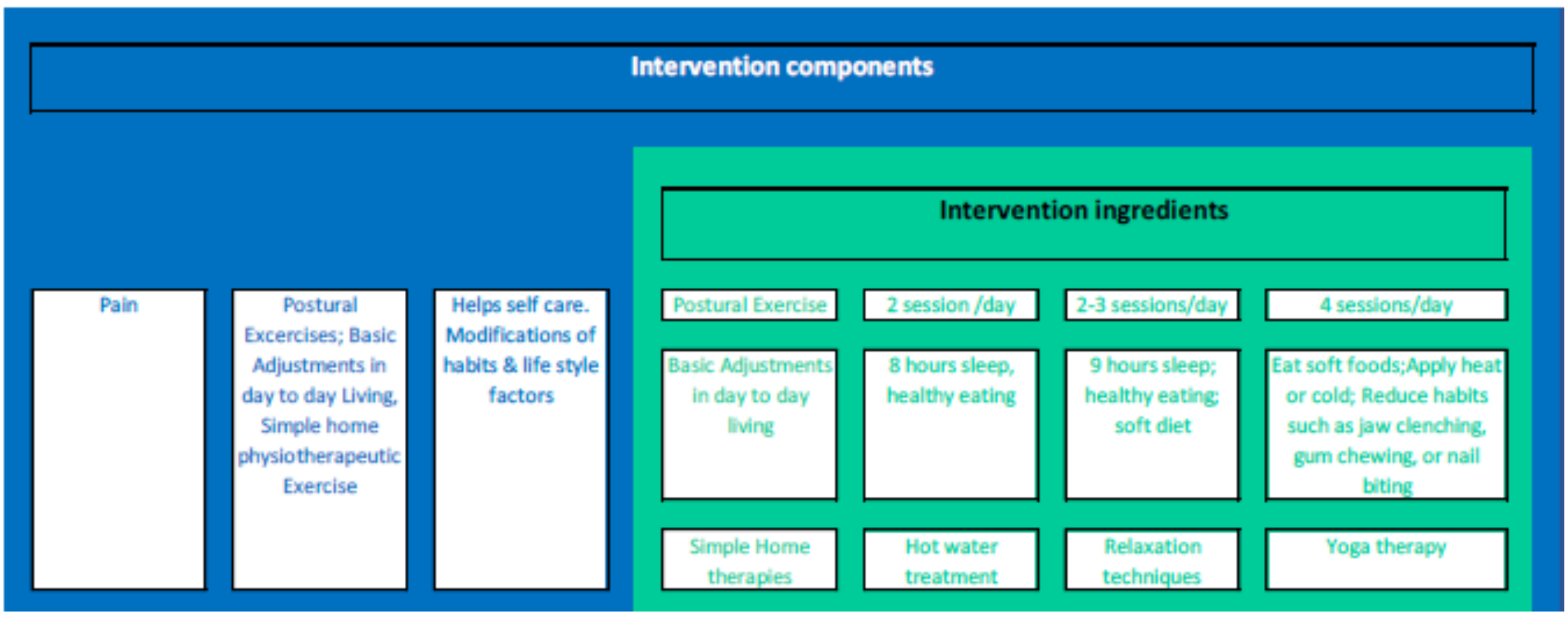

Figure 32 GUIDE-Rehab Programme 7 component 1 for Reversible Therapy Rehab

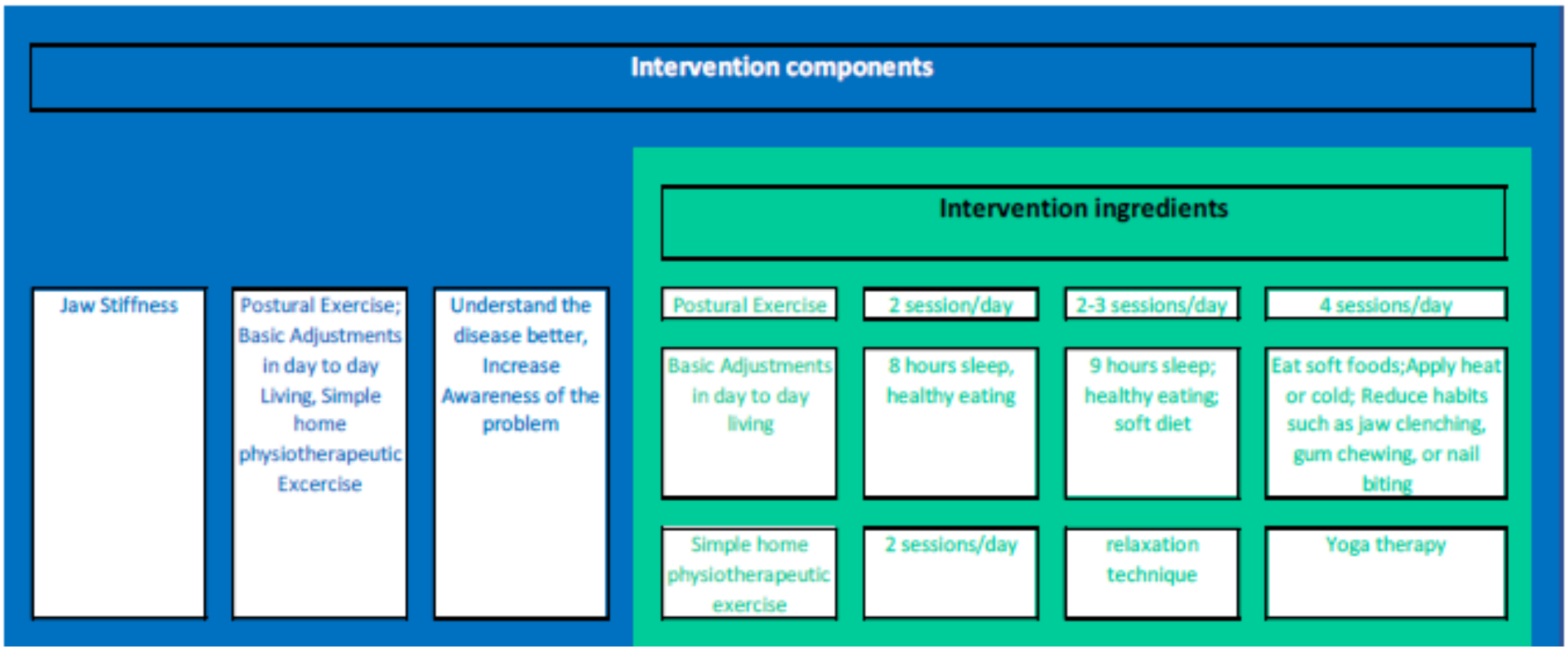

Figure 33 GUIDE-Rehab Programme 7 component 2 for Reversible Therapy Rehab

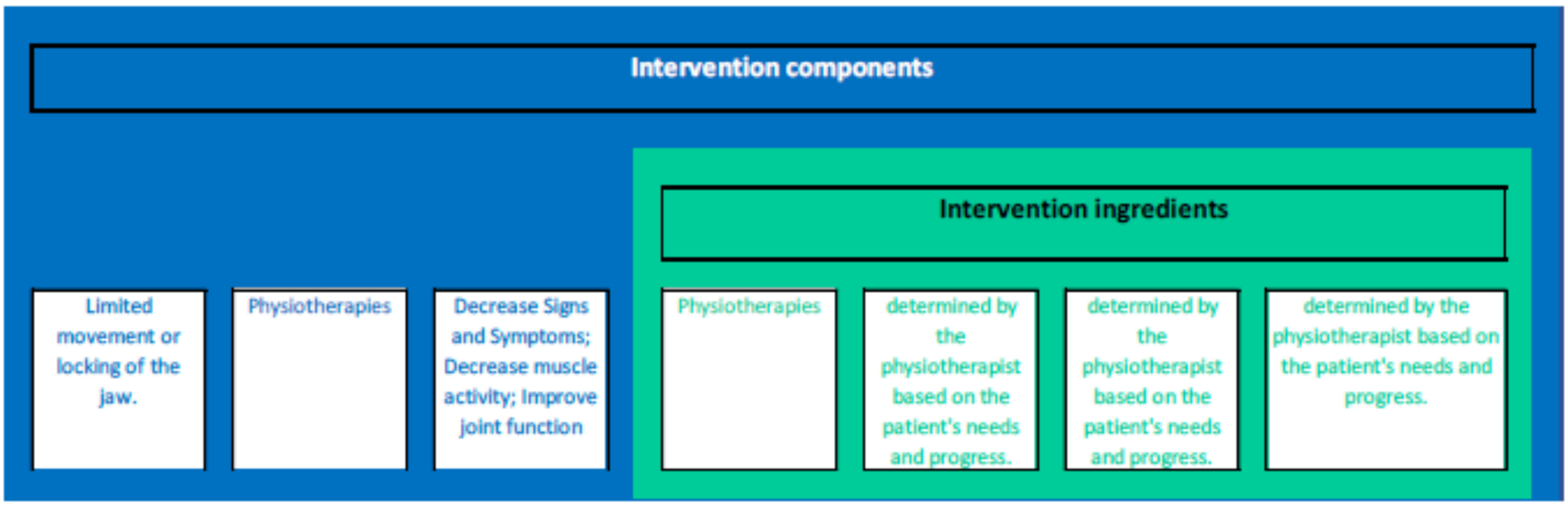

Figure 34 GUIDE-Rehab Programme 7 component 3 for Reversible Therapy Rehab

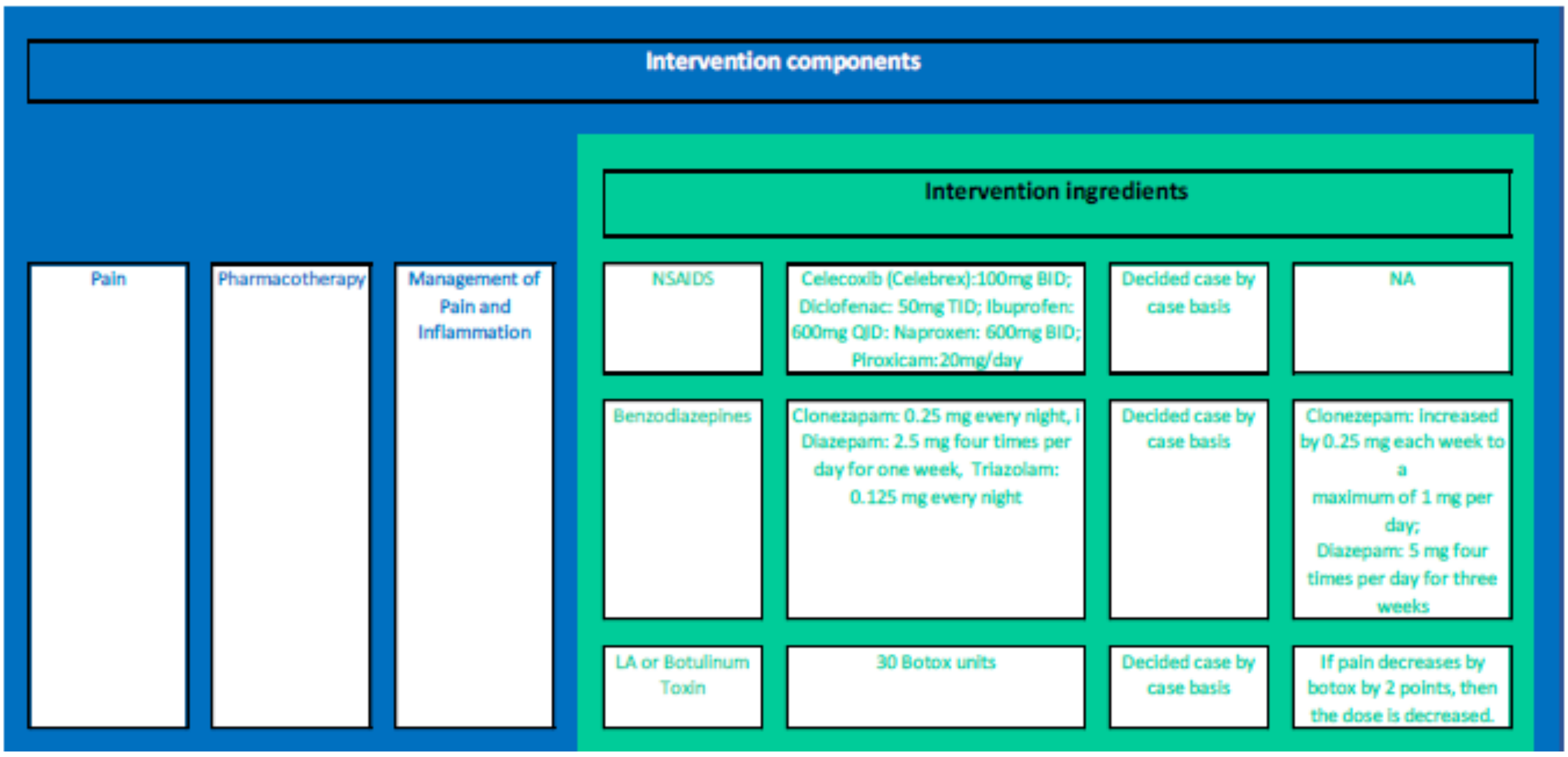

Figure 35 GUIDE-Rehab Programme 7 component 1 for Irreversible Therapy Rehab

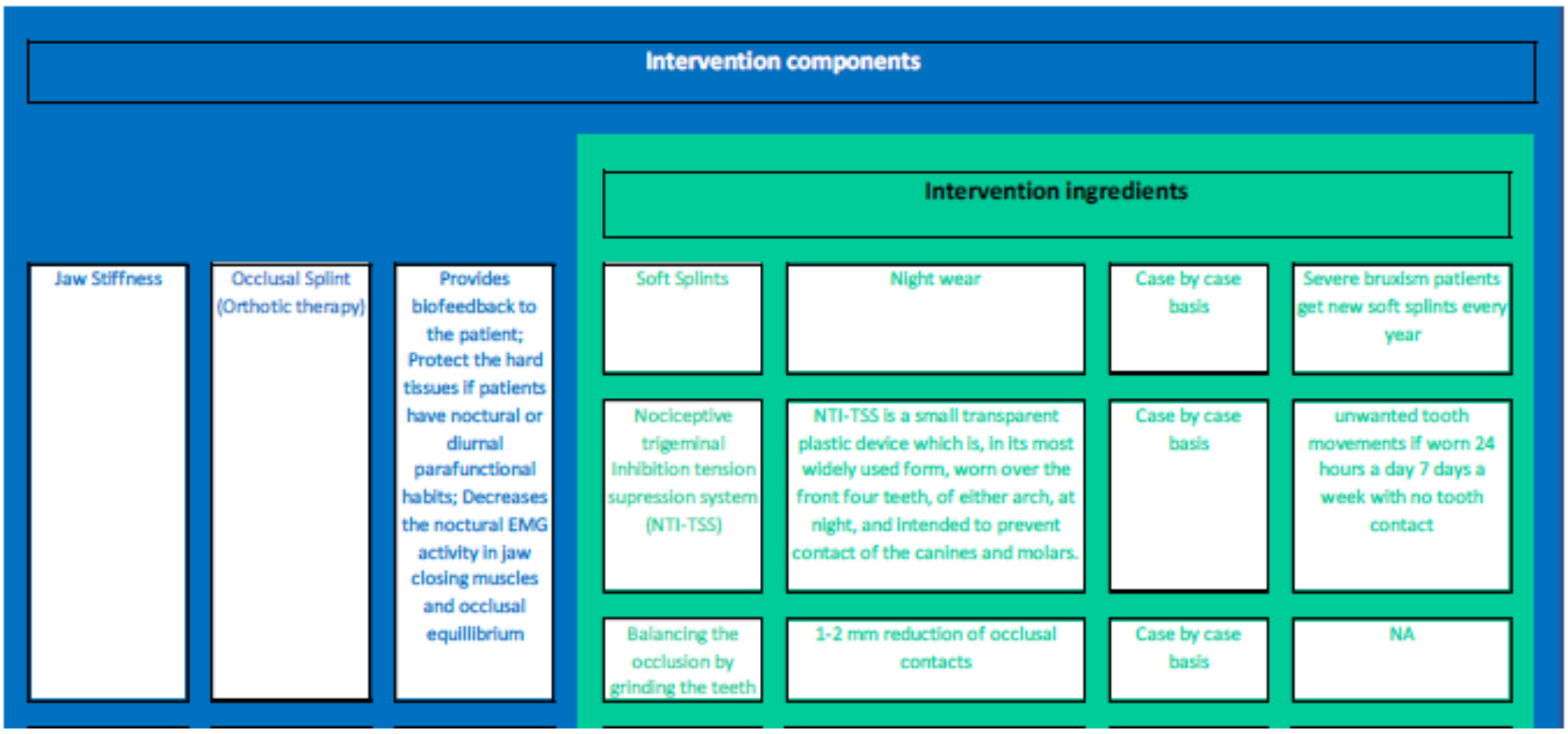

Figure 36 GUIDE-Rehab Programme 7 component 2 for Irreversible Therapy Rehab

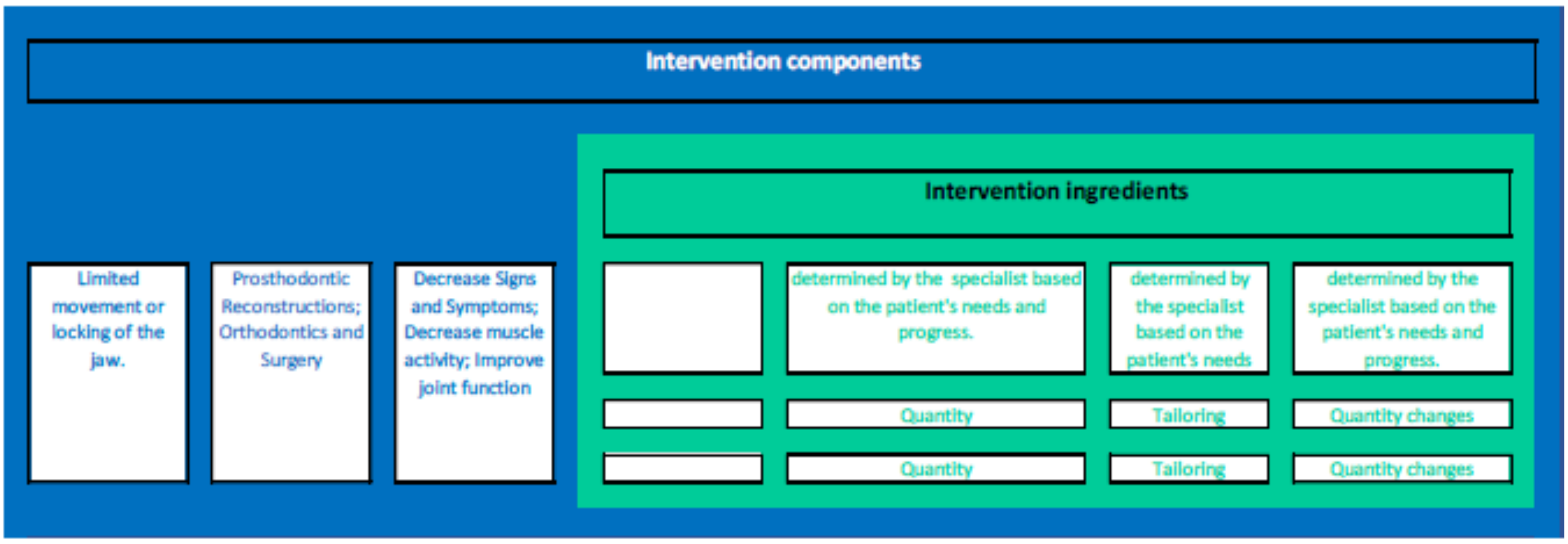

Figure 37 GUIDE-Rehab Programme 7 component 3 for Irreversible Therapy Rehab
